# Supplementary material for: C–N bond formation by a polyketide synthase
Source: Nat Commun. 2023 Mar 10;14:1319. doi: 10.1038/s41467-023-36989-w (PMC10006239; doi:10.1038/s41467-023-36989-w)
Supplement: Supplementary file 1 — Supplementary Information [file 41467_2023_36989_MOESM1_ESM.pdf]

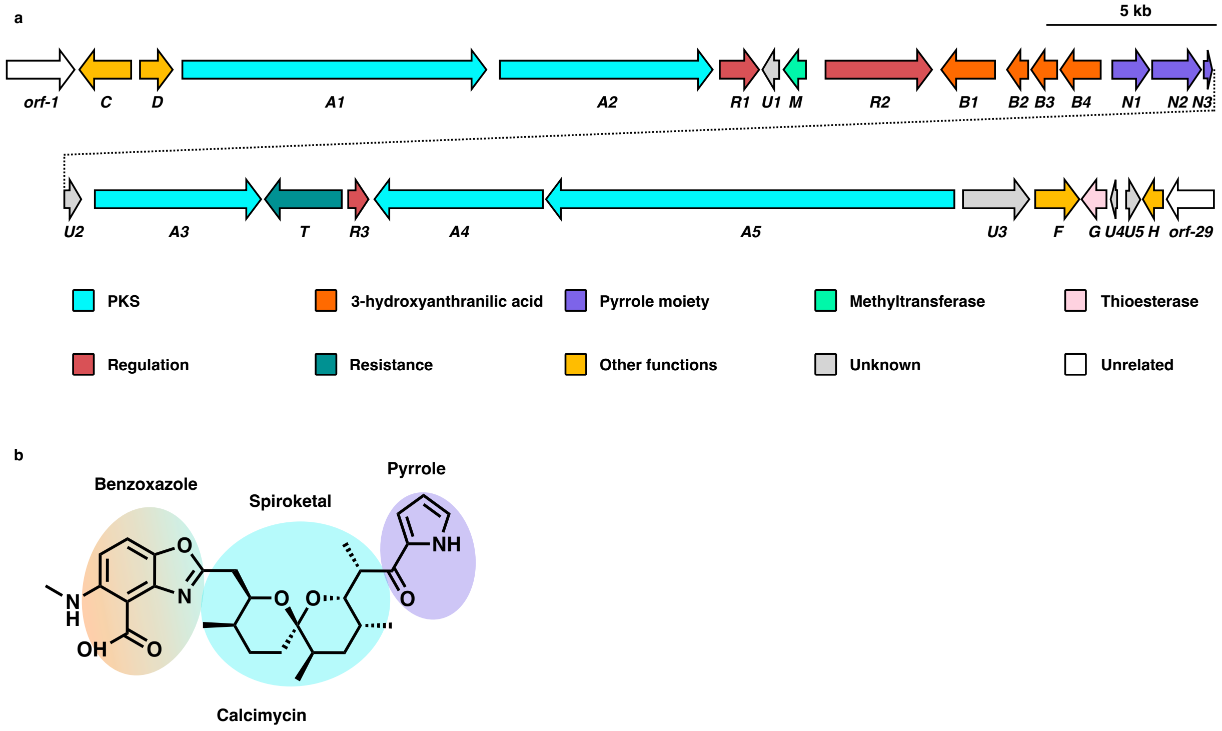

Supplementary Fig. 1 Calcimycin biosynthetic gene cluster in *Streptomyces chartreusis* NRRL 3882. a Open reading frames (ORFs) of the calcimycin biosynthetic gene cluster. The prefix of "cal" was omitted on purpose from each gene. **b** Structure of calcimycin.

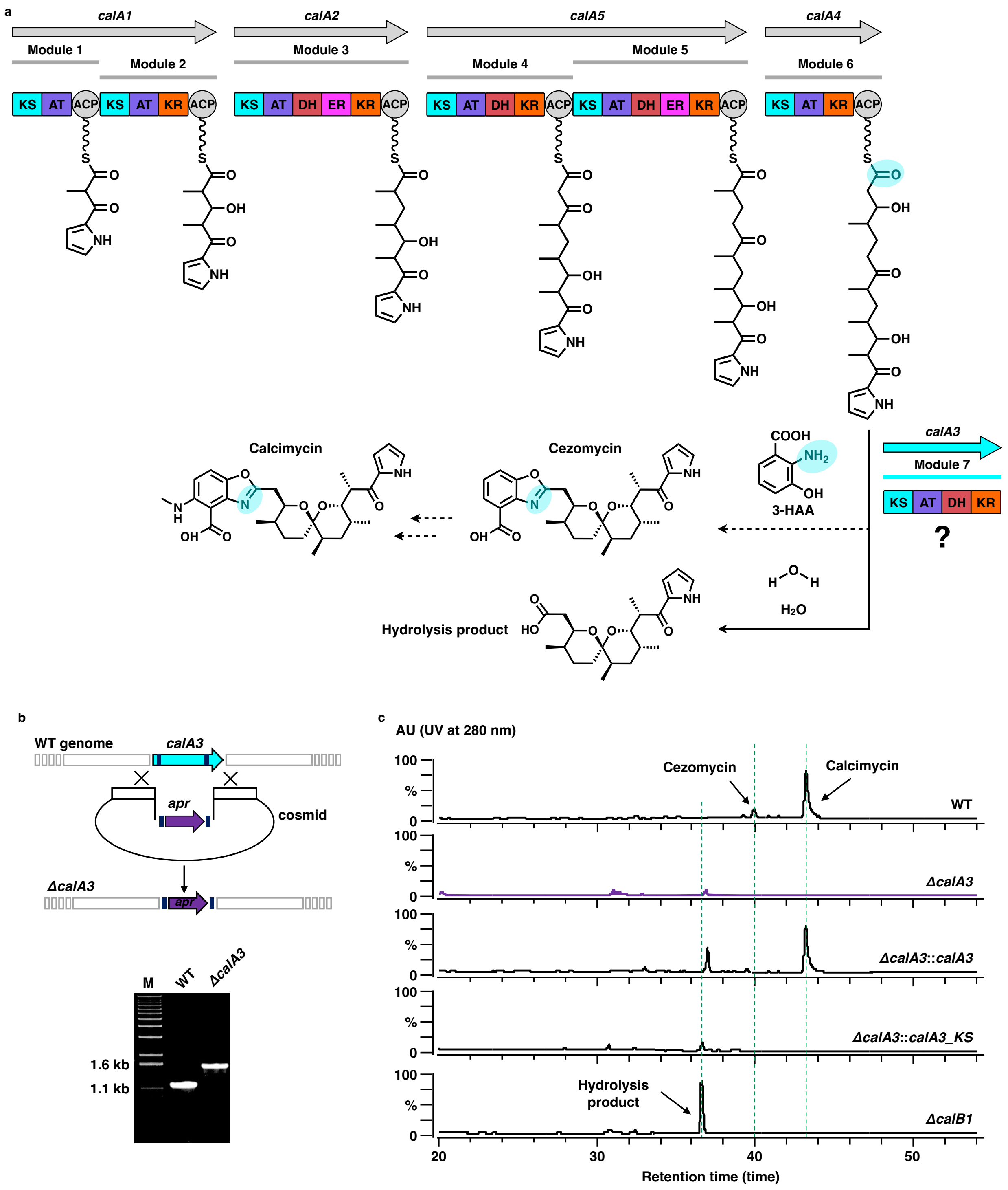

Supplementary Fig. 2 Proposed function of CalA3 in the biosynthesis of calcimycin. **a** The biosynthetic pathway by which CalA1, A2, A5, A4, and A3 generates calcimycin. The proposed chain-releasing function of CalA3 are indicated with a question mark. The C-N bond formed from upstream polyketide chain and 3-HAA are highlighted in cyan. **b** Gene knockout of *calA3* by using PCR-targeting strategy. *ΔcalA3* strain was generated

by double crossover replacement. The black rectangles indicate the positions of the primers used to generate the PCR products, which the identity was confirmed by a larger band in agarose gel electrophoresis, compared to that of the WT. At least three independent experiments were repeated with similar results. **c** HPLC traces comparison showing the necessity of *calA3* for calcimycin biosynthesis. *calB1* is responsible for the biosynthesis of substrate 3-HAA[1].

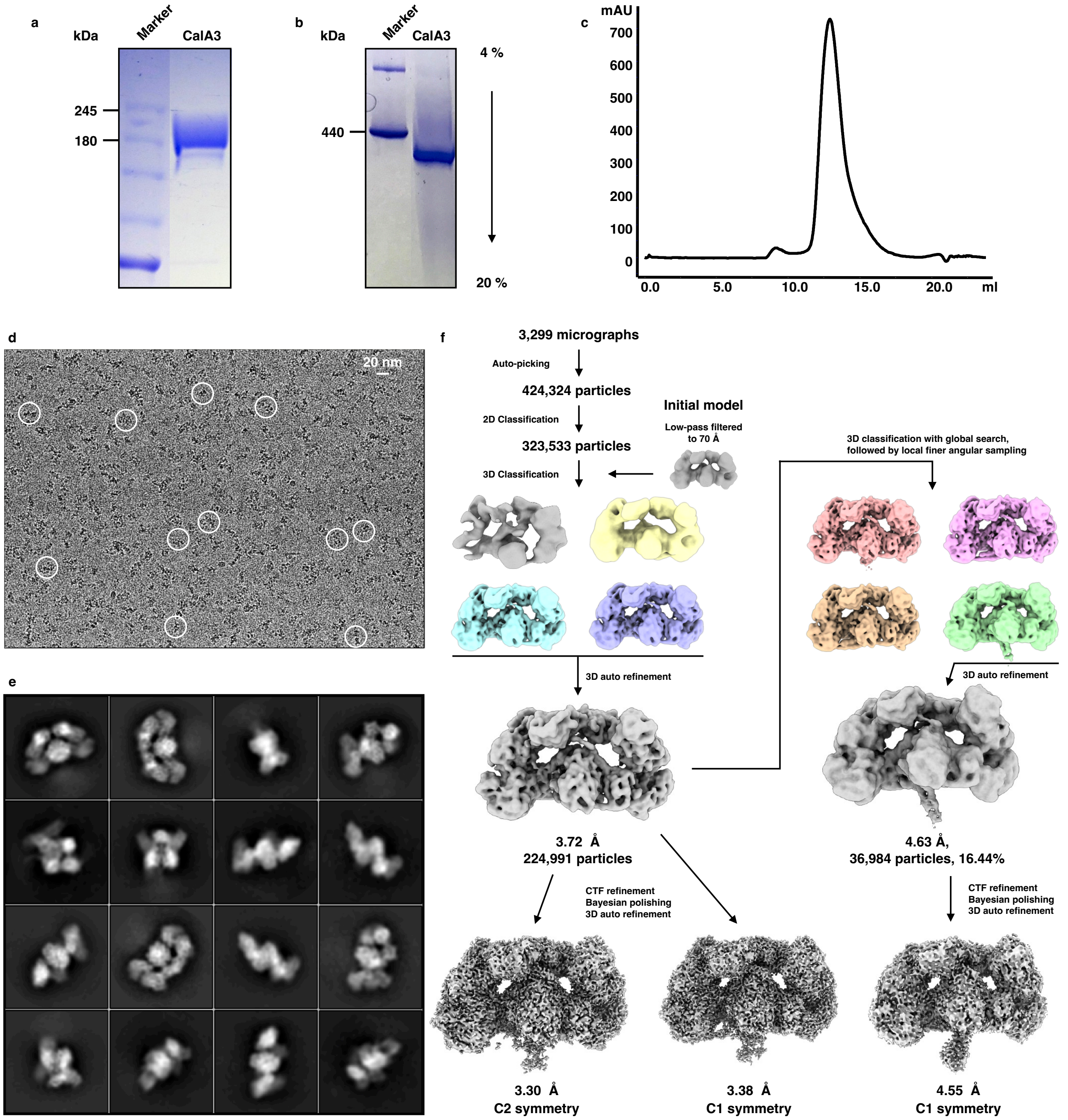

Supplementary Fig. 3 Purification and cryo-EM structure determination of CalA3. **a** SDS-PAGE analysis of purified CalA3. **b** Native-PAGE analysis of CalA3. A 4-20%

gradient was used. Source data are provided as a Source Data file (**a**-**b**). **c** Size exclusion chromatography (SEC, Superose 6 Increase) profile of CalA3. **d** One representative cryo-EM micrograph from the 3299 movie stacks of CalA3 with selected particles in white circles. Scale bar, 20 nm. **e** Representative 2D class averages. **f** A data processing workflow for the resolution-labeled cryo-EM maps. At least three independent experiments were repeated with similar results for each of **a**, **b** and **d**.

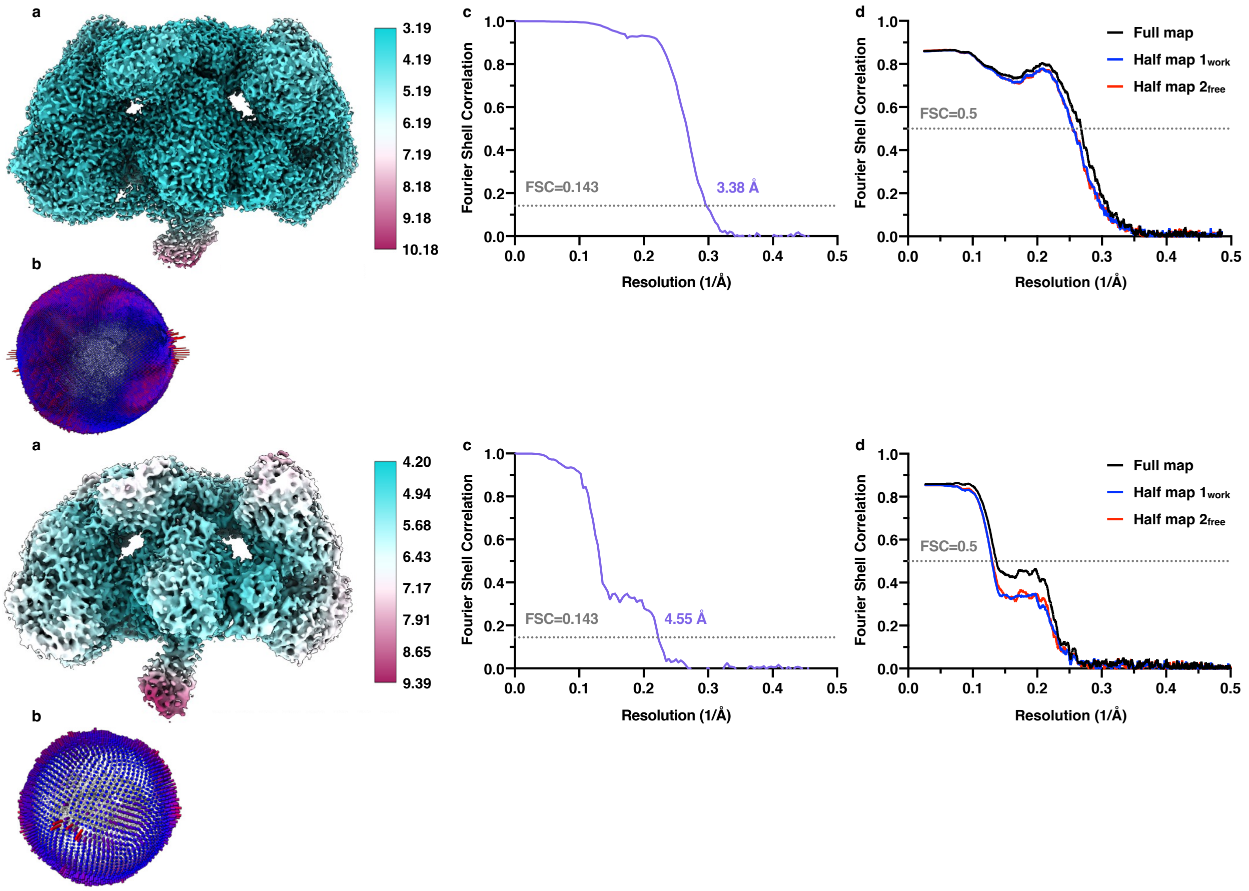

Supplementary Fig. 4 Cryo-EM analysis of CalA3 structures (related to Supplementary Fig. 3). Top, nearly horizontal docking domain (DD) conformation; bottom, nearly perpendicular DD conformation. **a** Local resolution of each map estimated in RELION. **b** Angular distribution of all particles used for the final reconstruction of each map. **c** Gold-standard FSC curves of the resolution-labeled cryo-EM map (FSC=0.143 criterion). **d** FSC curves of the final refined model versus the map that it was refined against (black); of the model refined in the first of the two independent maps used for the gold-standard FSC versus that same map (blue); and of the model refined in the first of the two independent maps versus the second independent map (red). The small difference between the work and free FSC curves indicates that the model did not suffer from overfitting.

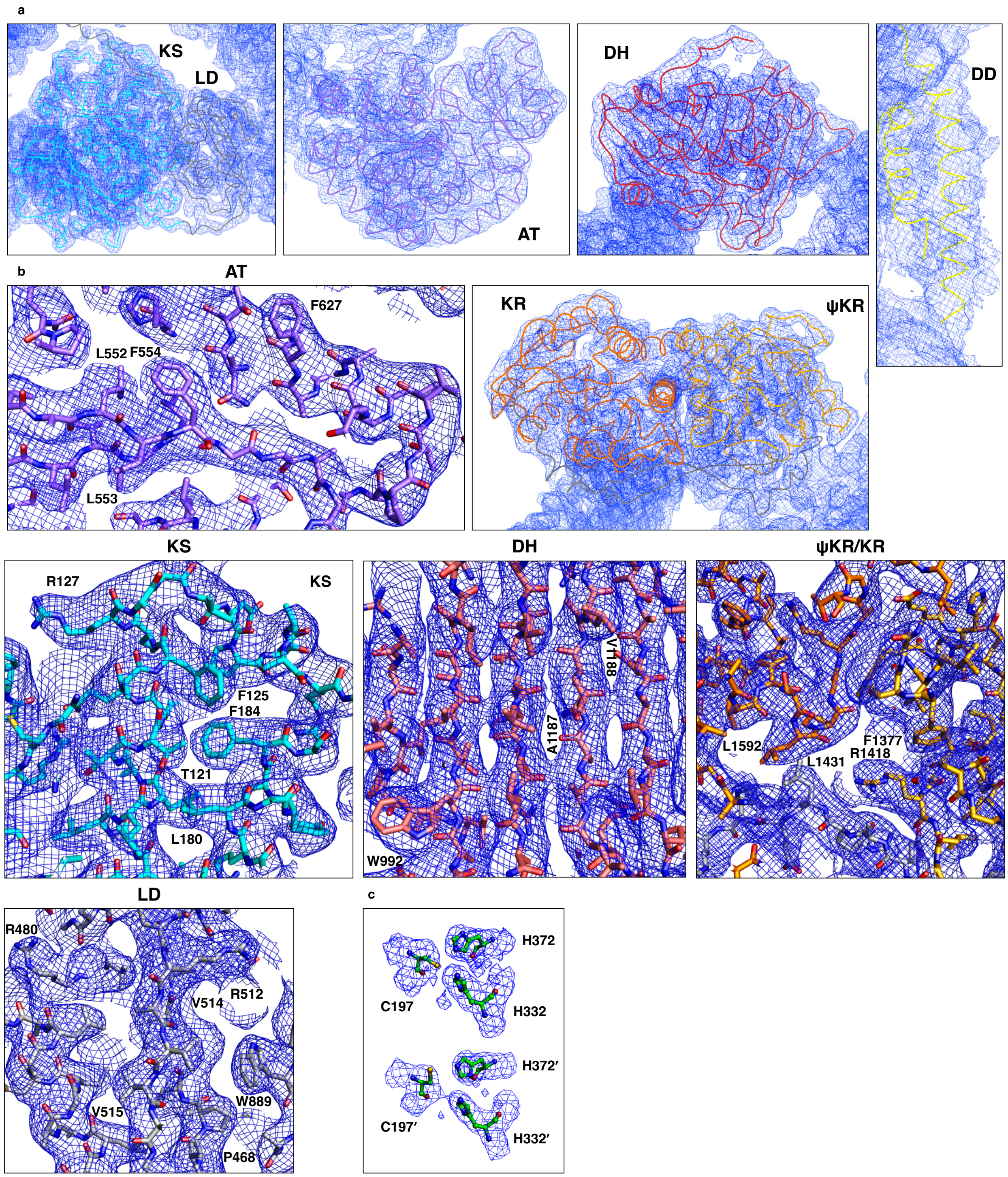

Supplementary Fig. 5 Representative cryo-EM map of the CalA3 domains. The displayed unsharpened map is from the 3.38 Å structure with C1 symmetry and shown as mesh, contoured at 0.011 (4.6σ) for the DH domain, 0.012 (5.0σ) for the KR, KS-LD and AT domains, and 0.003 (1.24σ) for the DD domain. Note that the DD model is merely docked in the map. The figure was generated in ChimeraX. **a** The cryo-EM map for each of the CalA3 domains, fitted with the cartoon represented atomic models (shown as lines).

b Close-up views of the regions of each domain, fitted with the full-atom represented atomic models (shown as sticks). **c** The DeepEMhancer[2]-enhanced cryo-EM map for each chain of the active site residues of KS domain. The carving distance is 3 Å. The map is contoured at 0.035 (0.9σ) for chain A and 0.085 (2.1σ) for chain B.

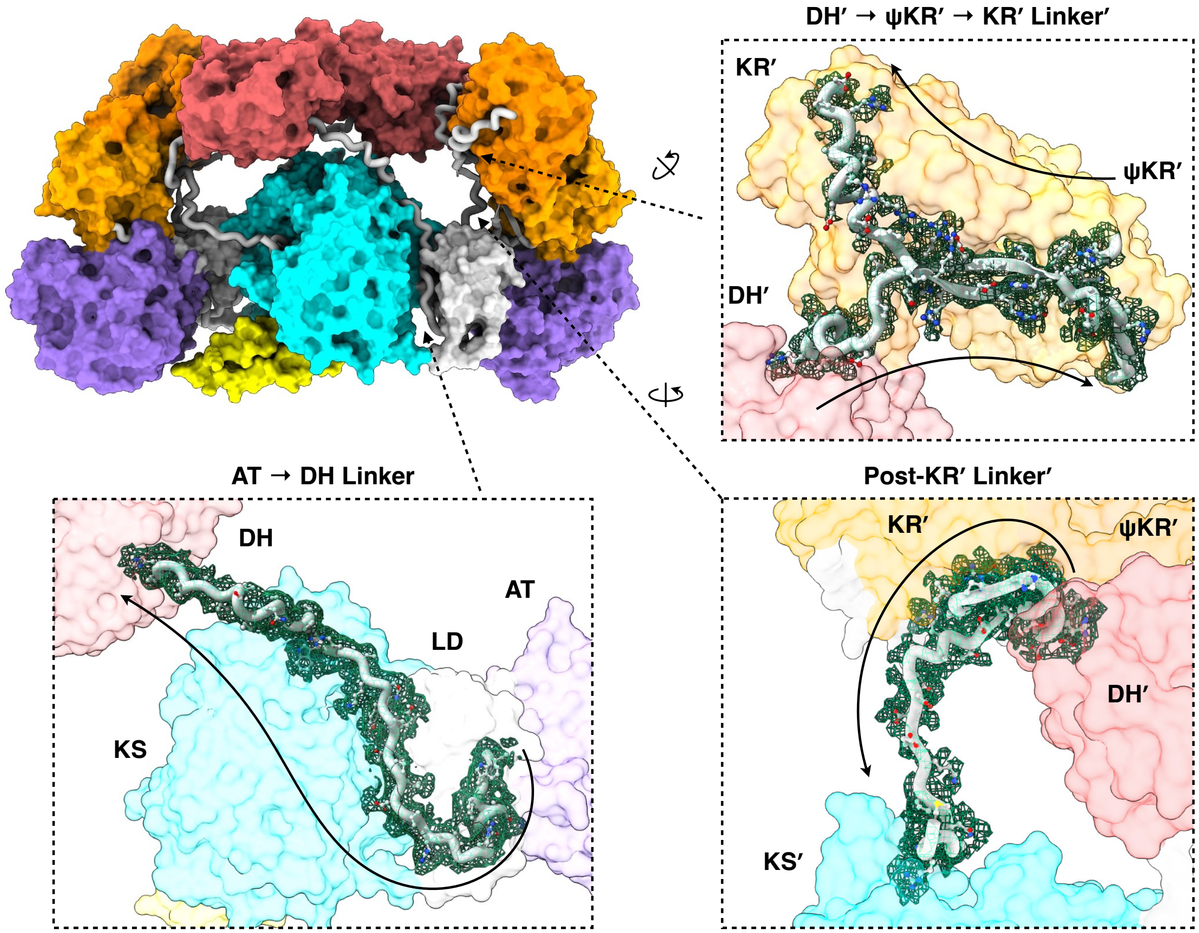

Supplementary Fig. 6 Linker-based domain organization of CalA3. Surface representation of CalA3, with domains colored as in Fig. 1. Interdomain linkers are highlighted as thick gray oval tubes. Sharpened Cryo-EM maps of linkers (unsharpened map for AT-DH linker) are fitted with corresponding atomic coordinates and shown in close-up views. The maps of AT-DH, DH'-ψKR'/KR' and post-KR' linkers are contoured at 0.0064 (2.65σ), 0.014 (2.76σ) and 0.010 (1.97σ), respectively.

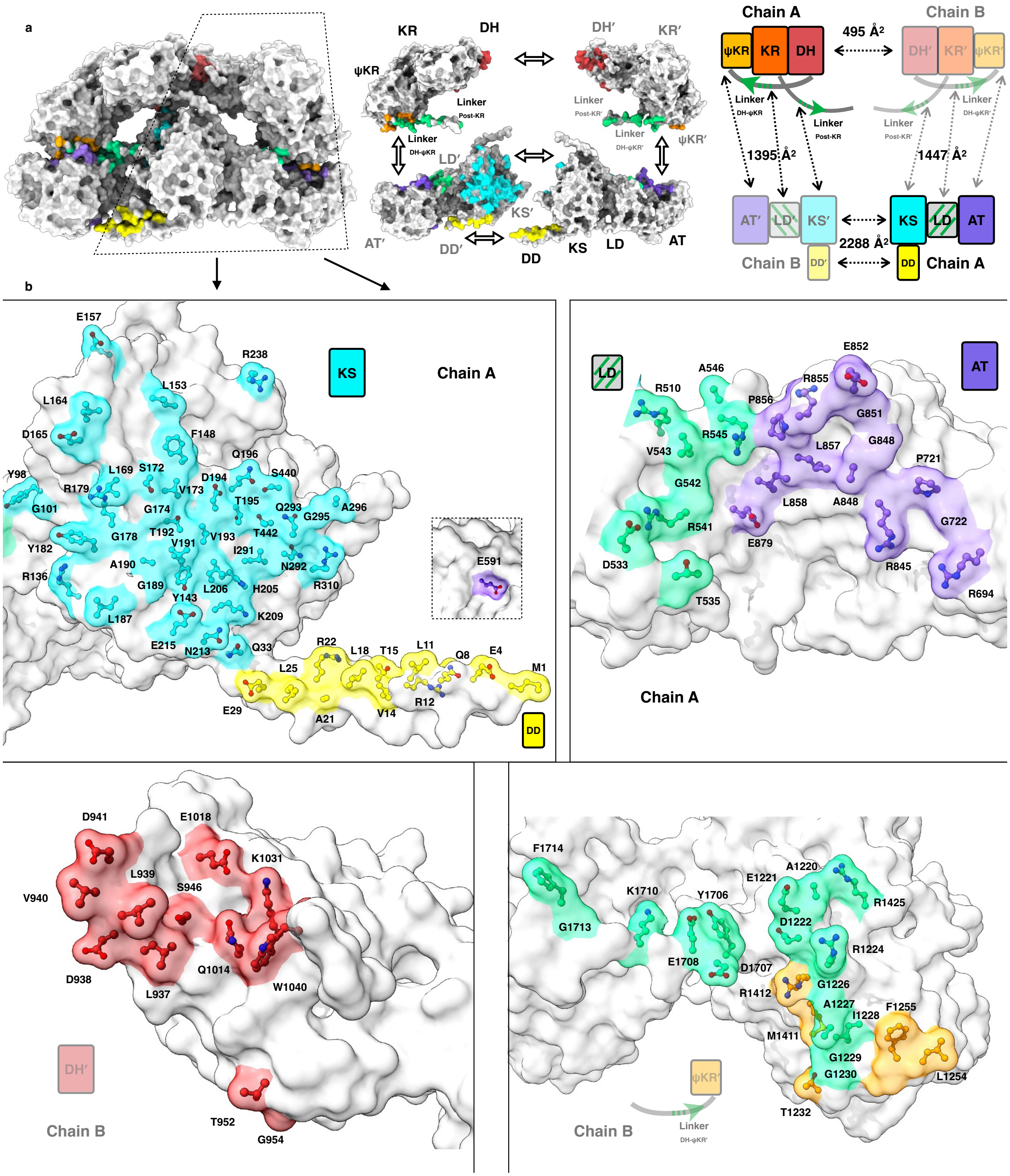

Supplementary Fig. 7 Interdomain interactions of CalA3. The interfaces mapped onto each domain are colored as in Fig. 1, except that of the linkers and LD are colored in green. **a** Overview of the interdomain interactions. Left, CalA3 structure shown as surface representation; Middle, split-up surface representation highlighting the interfaces contributed by each domain; Right, schematic diagram showing the buried surface area

in Å². **b** Interface residues of each domain are shown as sticks and colored accordingly. Only one side, bordered in (a) with dashed lines, is displayed.

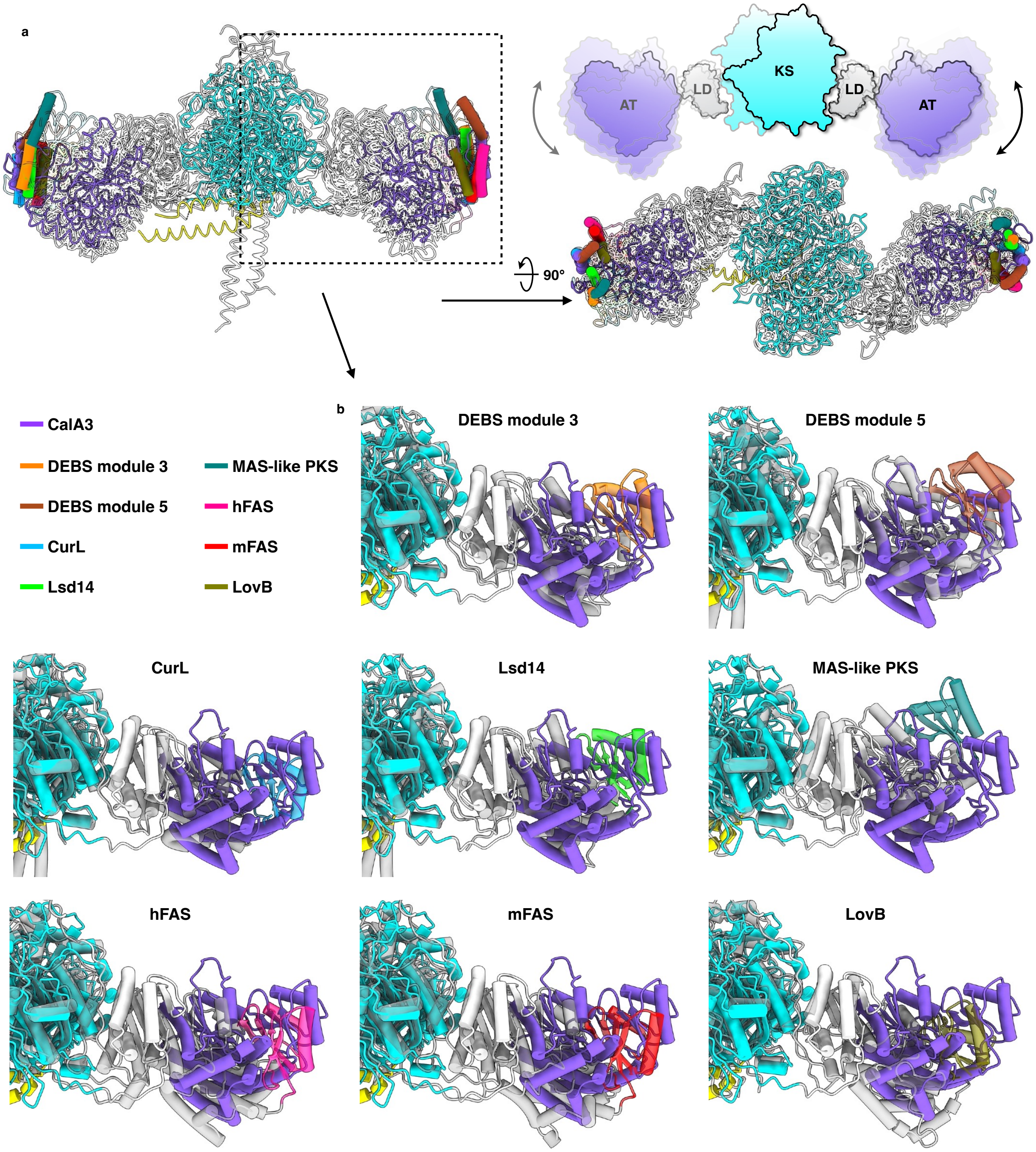

Supplementary Fig. 8 Structural comparisons of PKS and FAS condensing regions (KS-AT/MAT). a Overview of all the comparison structures superposed on the CalA3 KS dimer, shown in front and top views, and depicted as a schematic diagram. DD-KS-LD-AT domains of CalA3 are colored yellow, cyan, gray and purple, respectively. Other

structures are transparent with only a helix of AT shown in various colors to indicate the structural differences. **b** Individual superposition of CalA3 KS-AT structure with DEBS module 3 (PDB 2QO3)[3], DEBS module 5 (PDB 2HG4)[4], CurL (PDB 4MZ0)[5], Lsd14 (PDB 7S6B)[6], MAS-like PKS (PDB 5BP1)[7], hFAS (PDB 3HHD)[8], mFAS (PDB 2VZ8)[9], and LovB (PDB 7CPX)[10] condensing region structures. The small subdomain of AT (or MAT) in each homolog is colored differently.

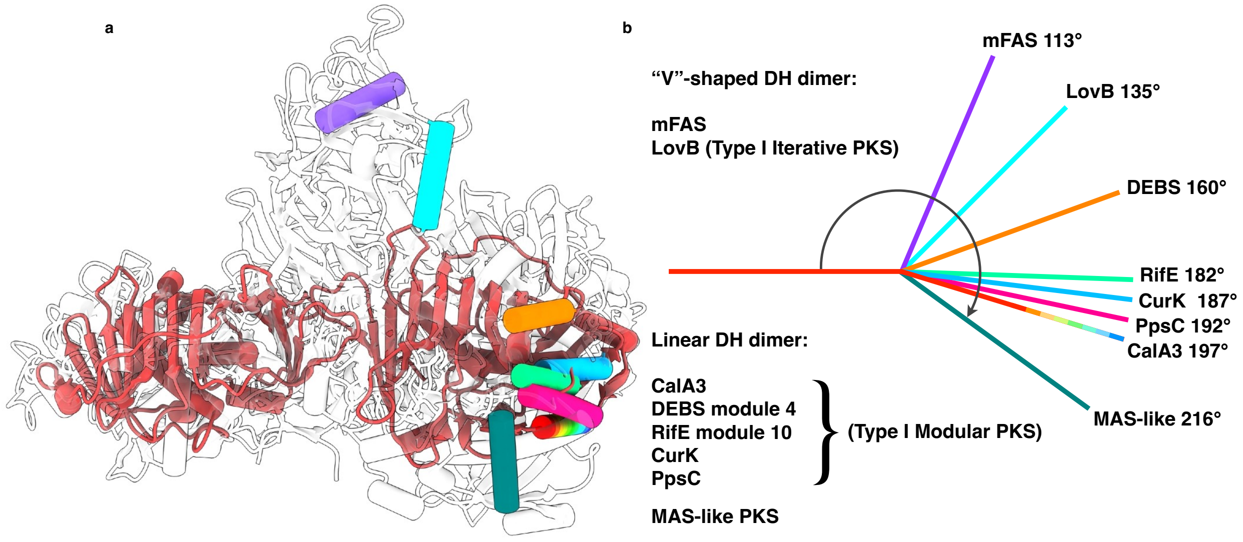

Supplementary Fig. 9 Dimeric DH domain organization of CalA3 compared to its homologs. a Superposition of CalA3 DH dimer with that of DEBS module 4 (PDB 3EL6)[11], RifE module 10 (PDB 4LN9)[12], CurK (PDB 3KG9)[13], PpsC (PDB 5I0K)[14], MAS-like PKS (PDB 5BP4)[7], mFAS (PDB 2VZ8)[9] and LovB (PDB 7CPX)[10] on one monomer. **b** Different interdomain angles illustrate the "V"-shaped or the linear DH dimer arrangements.

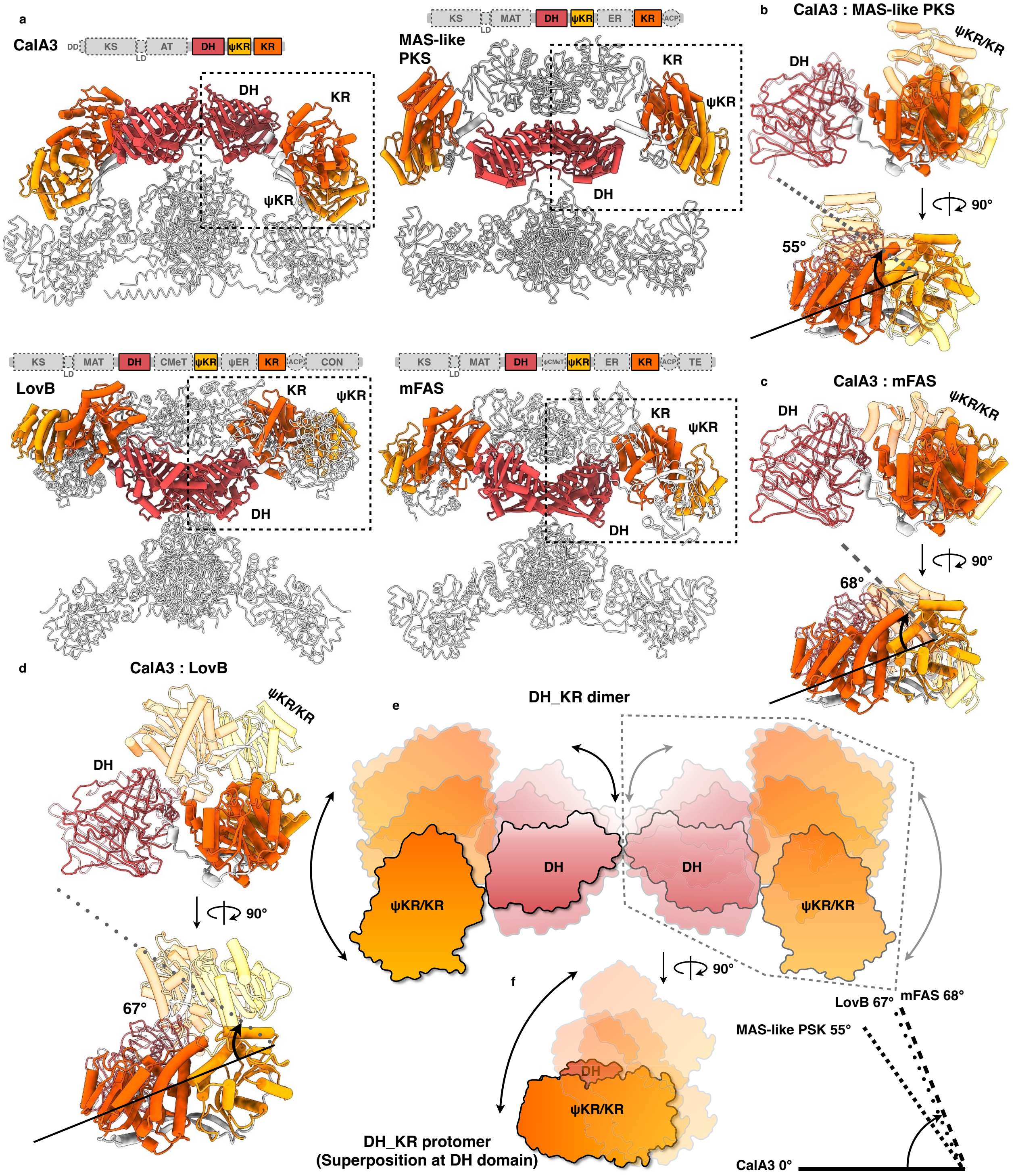

Supplementary Fig. 10 Structural comparisons of PKS and mFAS modifying regions (only DH-ψKR/KR considered). a Overview of CalA3, MAS-like PKS (PDB 5BP4)[7], mFAS (PDB 2VZ8)[9] and LovB (PDB 7CPX)[10] architectures, shown as line representations. The linear organization for each megaenzyme is shown above, with all the domains labeled. The DH and ψKR/KR domain dimers are highlighted in ribbon

representations and colored red and orange, respectively. **b-d** Individual superposition of CalA3 DH-ψKR/KR protomer on the DH domain. The relative angle between each ψKR/KR domain comparison illustrating the variable orientations. **e-f** Schematic diagrams revealing the various interdomain arrangements and relative angles, compared to CalA3 DH-ψKR/KR didomain.

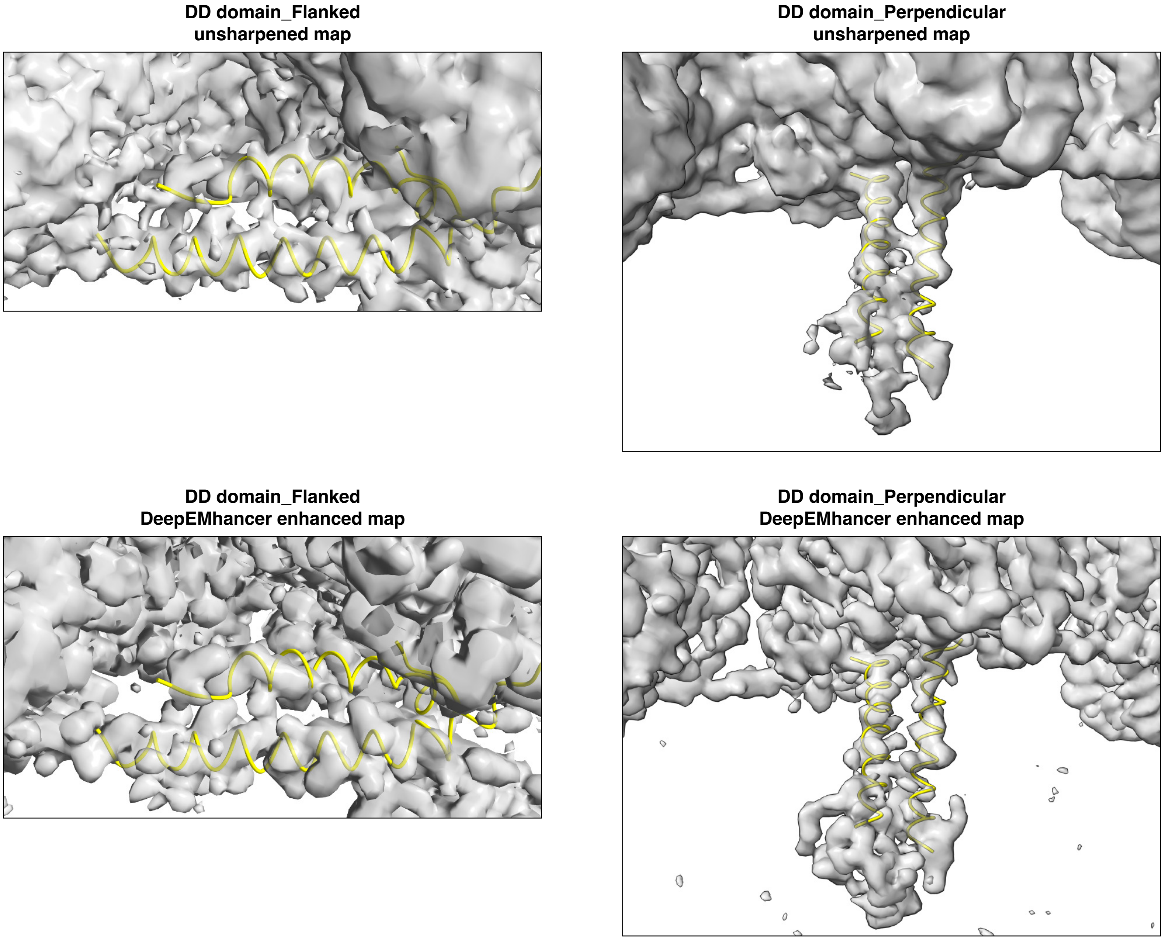

Supplementary Fig. 11 The cryo-EM maps of CalA3 N-terminal docking domains (DD). Left, flanked DD domain, unsharpened map contoured at 0.039 (1.6σ) and DeepEMhancer sharpened map contoured at 0.003 (0.07σ); right, perpendicular DD domain, unsharpened map contoured at 0.0067 (3.4σ) and DeepEMhancer sharpened map contoured at 0.09 (2.1σ).

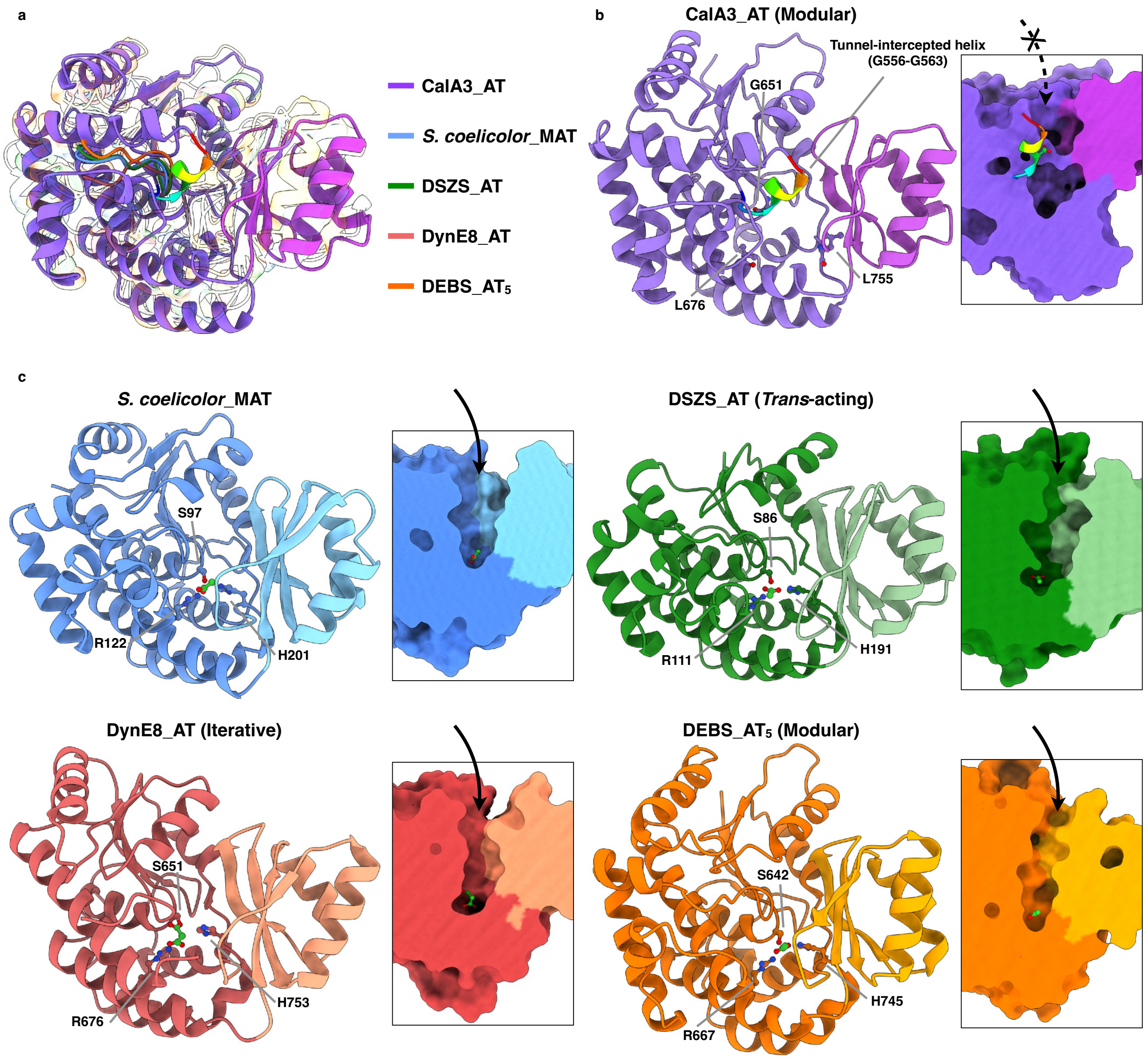

Supplementary Fig. 12 Structural comparisons of AT domains. a Superposition of inactive CalA3 AT domain with functional homologs. **b-c** Two subdomain-arranged AT structures of CalA3, *S. coelicolor* MAT (PDB 1NM2)[15], DSZS (PDB 3SBM)[16], DynE8 (PDB 4AMP)[17], and DEBS module 5 (PDB 2HG4)[4] are shown as ribbon and surface-clipped representations to reveal the individual architecture and substrate tunnel, respectively. Active site residues and substrates are shown as sticks. Substrate entrance of the tunnels are indicated by arrows. The tunnel-intercepting loop of CalA3 AT domain is rainbow colored in (b).

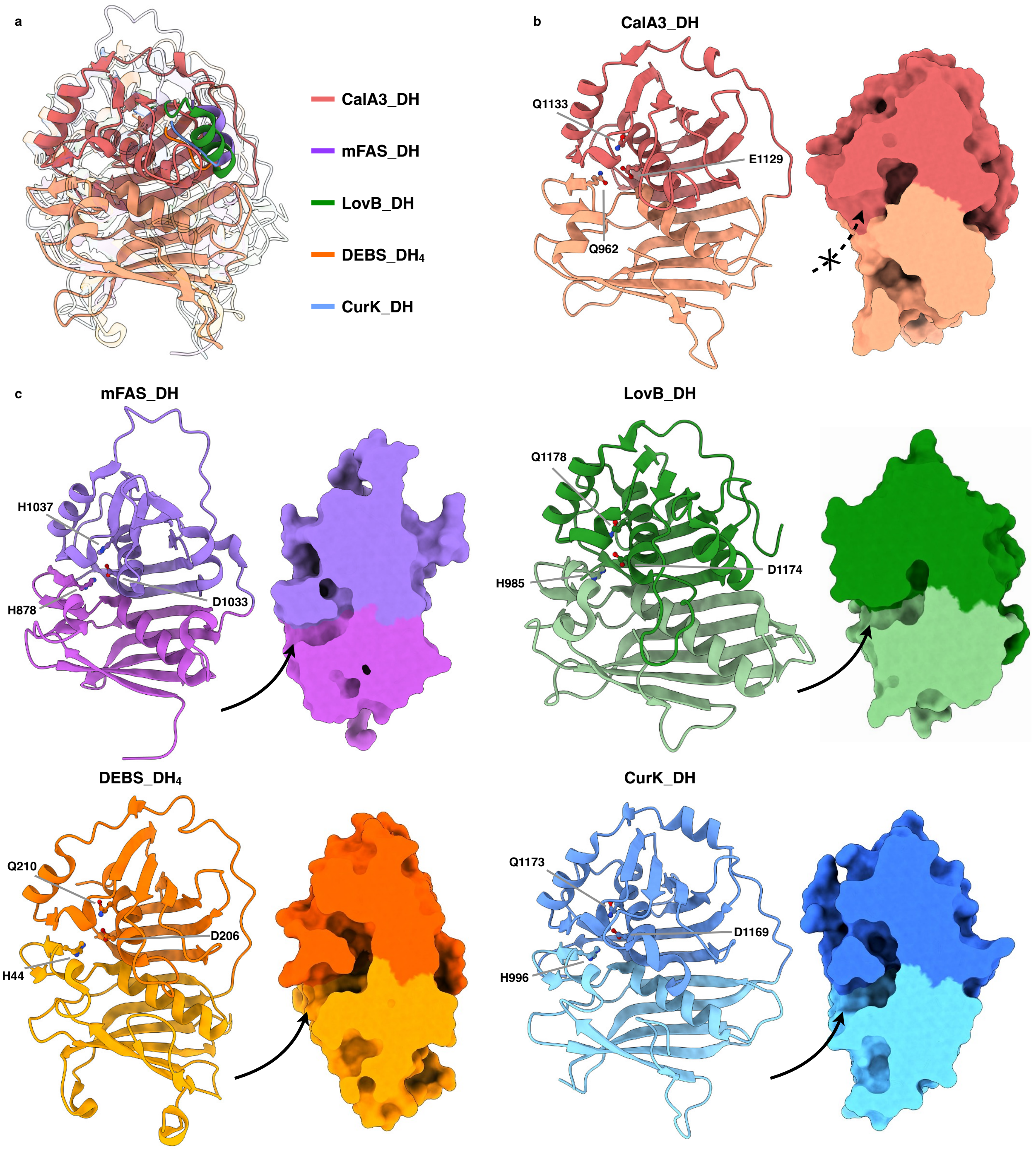

Supplementary Fig. 13 Structural comparisons of DH domains. a Superposition of inactive CalA3 DH domain with functional homologs. **b-c** Two hotdog fold-contained DH structures of CalA3, mFAS (PDB 2VZ8)[9], LovB (PDB 7CPX)[10], DEBS module 4 (PDB 3EL6)[11] and CurK (PDB 3KG9)[13] are shown as ribbon and surface-clipped representations to reveal the individual architecture and substrate tunnel, respectively. Active site residues are shown as sticks. Substrate entrance of the tunnels are indicated by arrows.

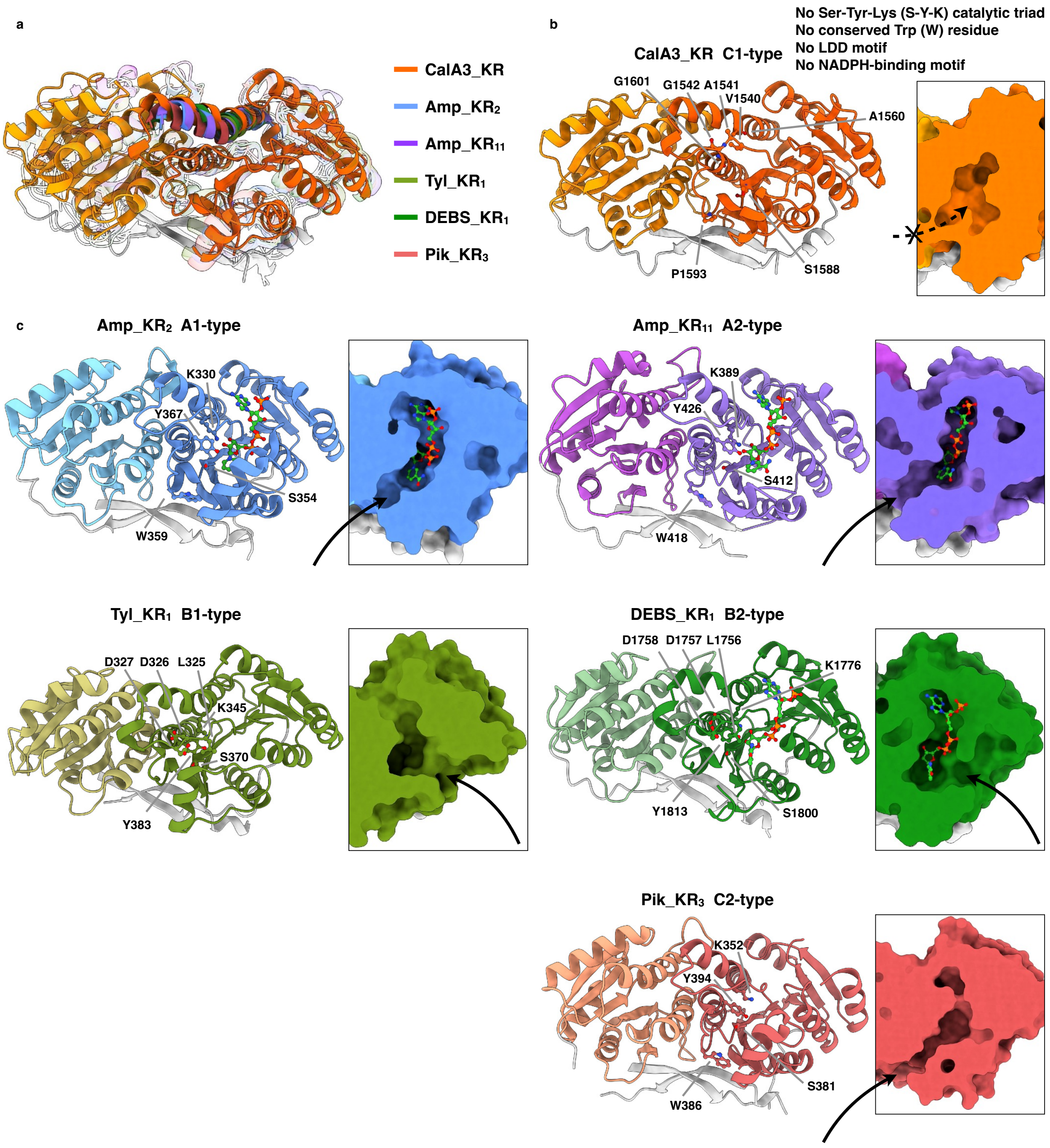

Supplementary Fig. 14 Structural comparisons of different types of ψKR/KR domains. a Superposition of inactive CalA3 ψKR/KR domain with functional homologs. **b-c** ψKR/KR structures of CalA3 (C1-type), Amp module 2 (A1-type, PDB 3MJS)[18], Amp module 11 (A2-type, PDB 4L4X)[19], Tyl module 1 (B1-type, PDB 2Z5L)[20], DEBS module 1 (B2-type, PDB 2FR1)[21] and Pik module 3 (C2-type, PDB 3QP9)[22] are shown as ribbon and surface-clipped representations to reveal the individual architecture and substrate tunnel, respectively. The S-Y-K active site residues are shown as sticks. Featured structural elements for differentiation between each type of KR: the conserved W residue

for the A1, A2 and C2 types; the LDD motif for the B1 and B2 types; and two different substrate entrances for the A and B types.

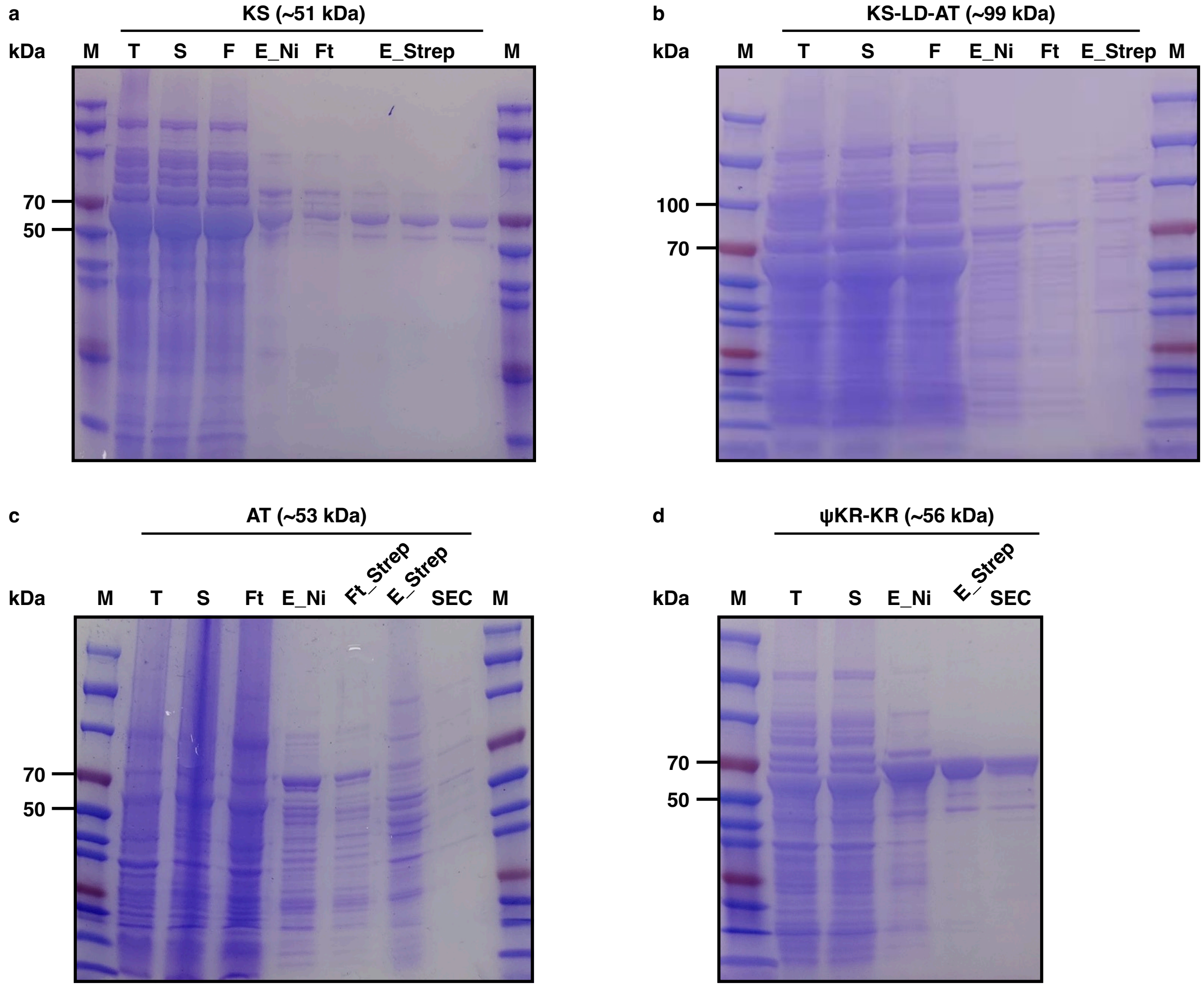

Supplementary Fig. 15 SDS-PAGE analysis of truncated versions of CalA3. The targeted theoretical molecular weight of each truncation is labeled in **a**, KS domain; **b**, KS-LD-AT domain; **c**, AT domain; **d**, ψKR/KR domain. Note that only ψKR/KR domain is successfully purified. At least three independent experiments were repeated with similar results for each of **a**, **b**, **c** and **d**. Source data are provided as a Source Data file. Abbreviations used for each gel: M, marker; T, total protein; S, supernatant; Ft, flow through; E_Ni, elution of His-tagged protein; E_Strep, elution of StrepII-tagged protein, SEC, size exclusion chromatography.

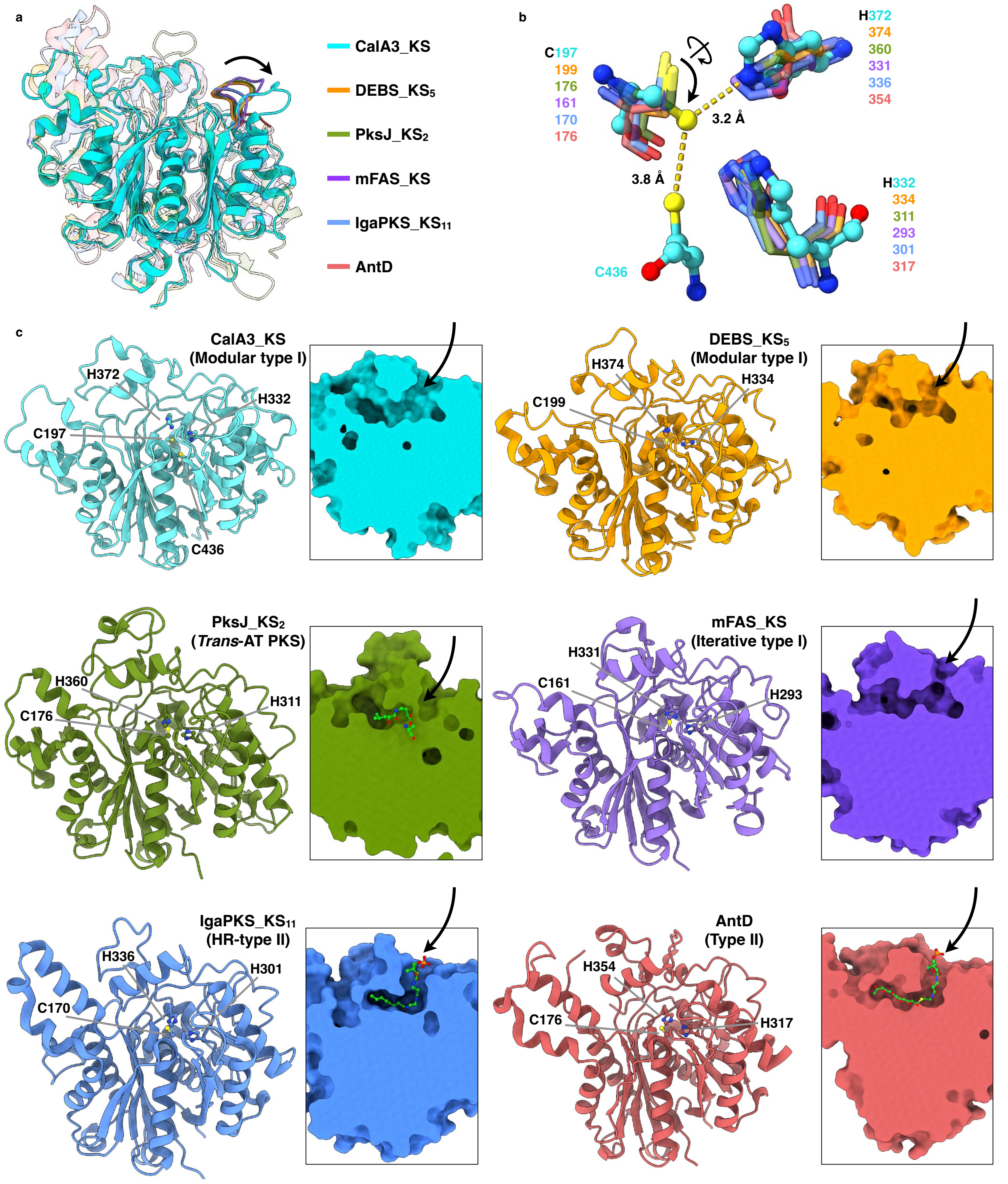

Supplementary Fig. 16 Structural comparisons of KS domains. **a** Superposition of CalA3 KS domain with homologs. A notably shifted loop is indicated by an arrow. **b** Superposition of the conserved active site residues of KS domains. Compared to the homologs, the active site residues of CalA3 are shown in balls and sticks. The rotated C197 is marked by an arrow, and its distance between H372 and C436 are labeled. **c** KS

domain structure of CalA3, DEBS module 5 (PDB 2HG4)[4], PksJ module 2 (PDB, 4NA2)[23], mFAS (PDB 2VZ8)[9], IgaPKS_KS11 (PDB 6KXF)[24], and AntD (PDB 6SMP)[25] are shown as ribbon and surface-clipped representations to reveal the individual architecture and substrate tunnel, respectively. Catalytic triads and polyketide substrates are shown as sticks. Substrate entrance of the tunnels are indicated by arrows.

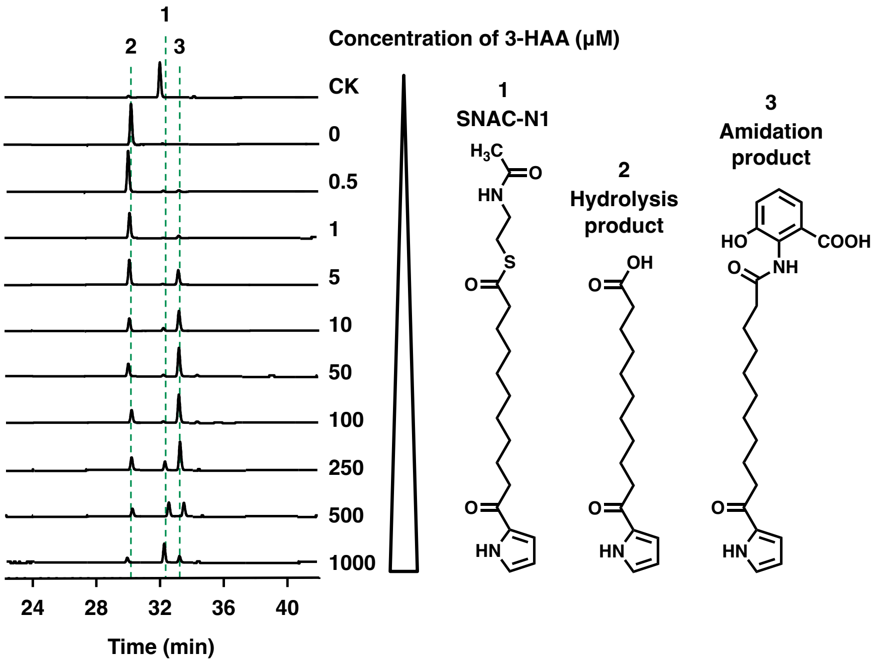

Supplementary Fig. 17 Competition experiment of CalA3-catalyzed hydrolysis and amidation. A series of HPLC profile at the gradually increasing concentrations of 3-HAA.

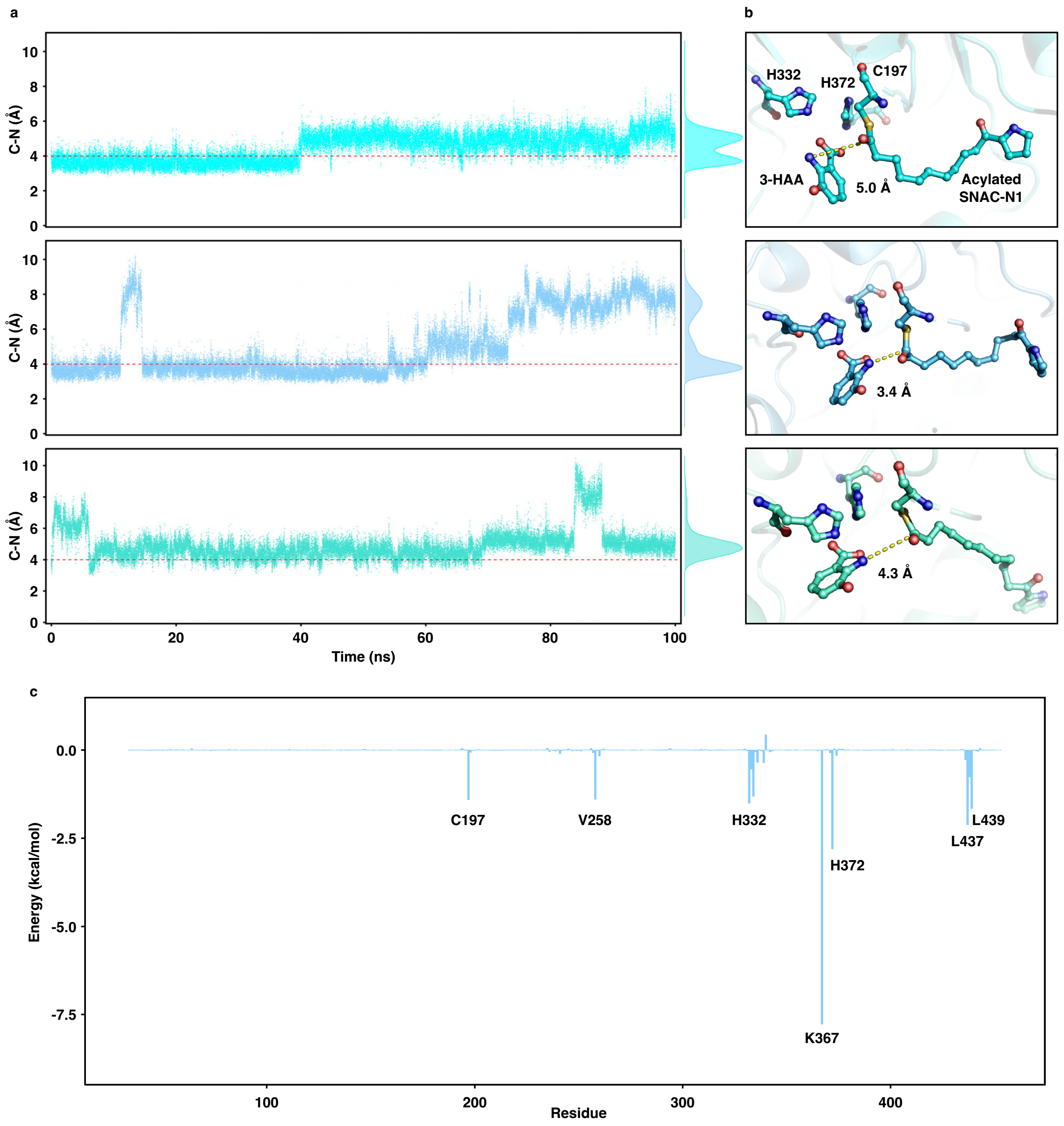

Supplementary Fig. 18 Molecular dynamics simulations of the interactions between substrates and active site residues. a The distance variation between C_α of C197-acylated SNAC-N1 and N of 3-HAA over time of 100 ns. Three independent repeats were performed. **b** Main cluster structure of each trajectory. **c** Molecular mechanics/Generalized born surface area (MM/GBSA) binding energy decomposition shows that the critical residues of H332, K367, H372, L437 and L439 contribute 1.5, 7.7, 2.8, 2.1, 1.7 kcal/mol, respectively. C197 and V258 both contribute 1.4 kcal/mol.

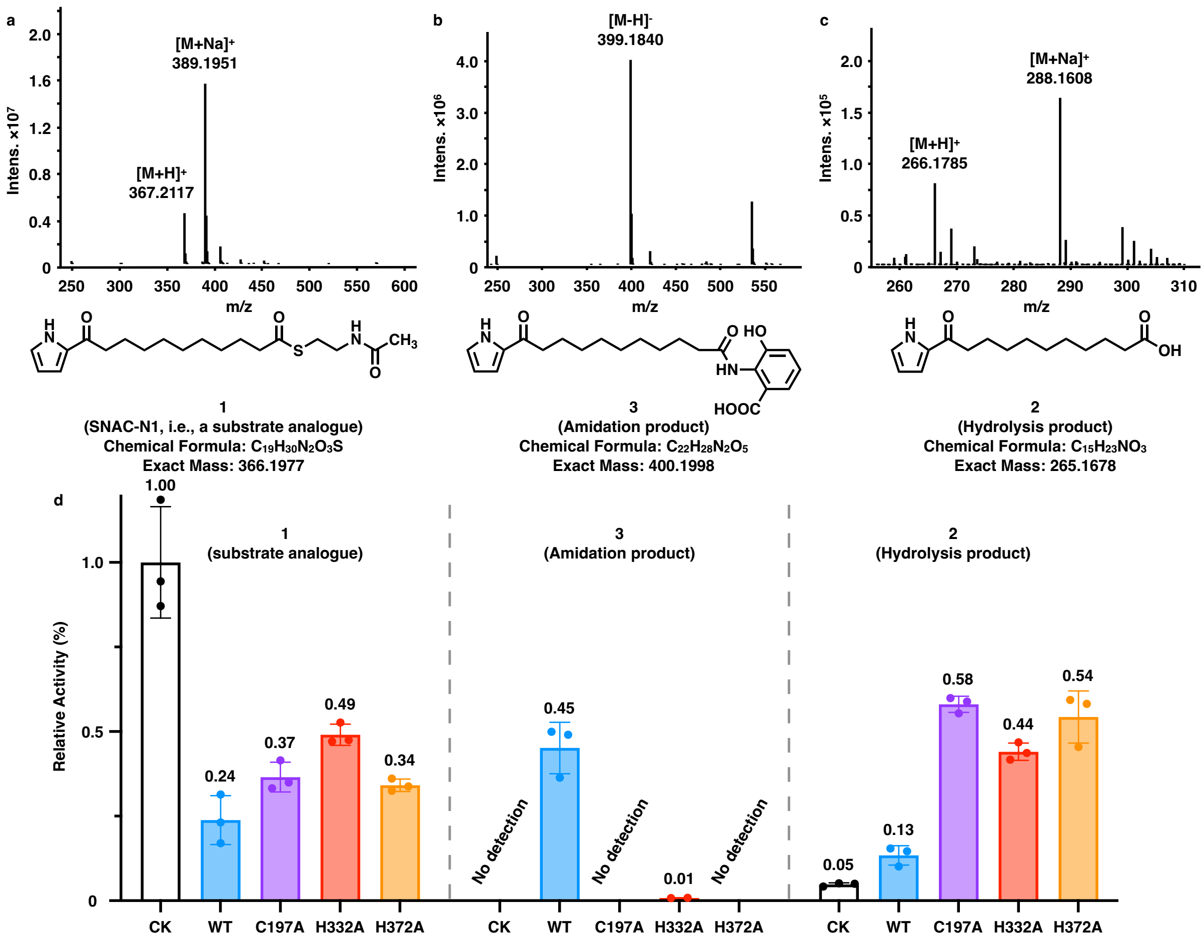

Supplementary Fig. 19 Activity assay of CalA3 WT and mutants in the KS domain. a-c Representative mass spectra of compound 1, 3, and 2 in positive, negative, and positive ion mode, respectively. Compound 1 has calculated and experimentally determined m/z $[M+H]^+$ values of 367.1977 and 367.2117; compound 3 has calculated and experimentally determined m/z $[M-H]^-$ values of 399.1998 and 399.1840; compound 2 has calculated and experimentally determined m/z $[M+H]^+$ values of 266.1678 and 266.1785. **d** The plot shows relative activities of compound 1, 3 and 2 of WT and the active site residue mutants in the KS domain (as % of the average peak area of compound 1 in the control). Data are presented as mean values +/- SD from three biologically independent experiments (*n*=3). Source data are provided as a Source Data file.

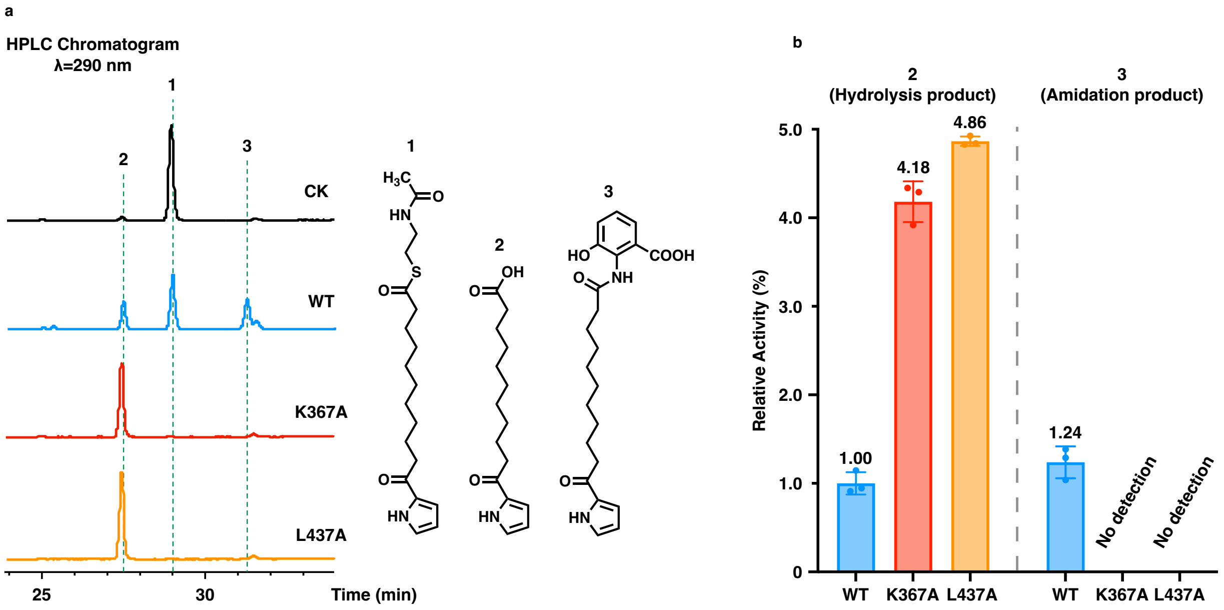

Supplementary Fig. 20 Activity assay of CalA3 mutants of positioning residues in the KS domain. a HPLC traces at λ=290 nm showing the products of the *in vitro* reactions catalyzed by CalA3 WT or positioning residue mutants. **b** The plot shows relative activities of compound 2 and 3 of WT and the positioning residue mutants in the KS domain (as % of the average peak area of compound 2 in the WT). Data are presented as mean values +/- SD from three biologically independent experiments (*n*=3). Source data are provided as a Source Data file.

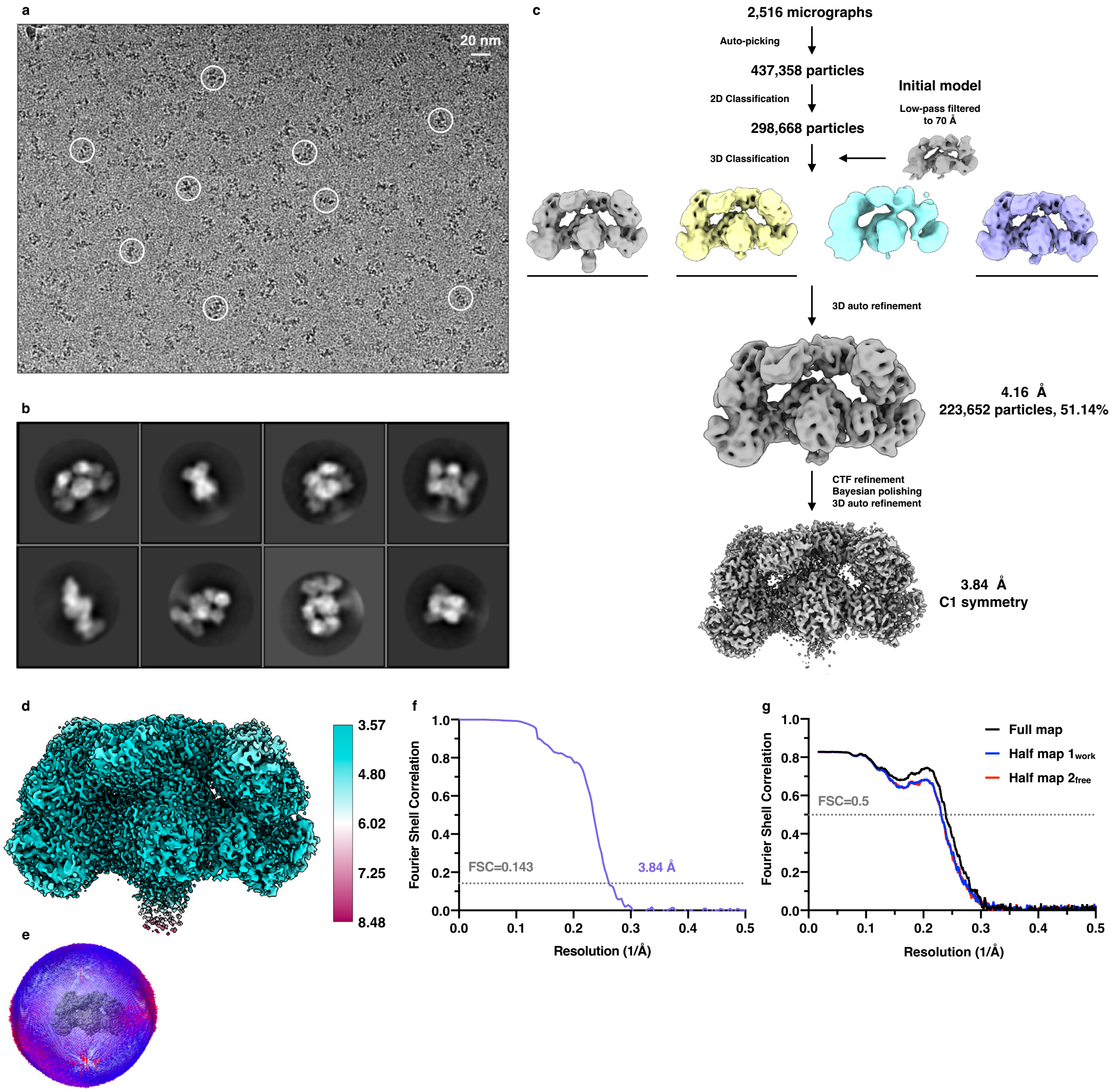

Supplementary Fig. 21 Cryo-EM structure determination of CalA3 with the amidation product. **a** One representative cryo-EM micrograph from the 2516 movie stacks of CalA3 with selected particles in white circles. Scale bar, 20 nm. At least three independent experiments were repeated with similar results. **b** Representative 2D class averages. **c** A data processing workflow for the resolution-labeled cryo-EM maps. **d** Local resolution of the map estimated in RELION. **e** Angular distribution of all particles used for the final reconstruction of the map. **f** Gold-standard FSC curves of the resolution-labeled cryo-EM map (FSC=0.143 criterion). **g** FSC curves of the final refined model versus the map that it was refined against (black); of the model refined in the first of the two independent maps used for the gold-standard FSC versus that same map (blue); and of the model refined in the first of the two independent maps versus the second independent

map (red). The small difference between the work and free FSC curves indicates that the model did not suffer from overfitting.

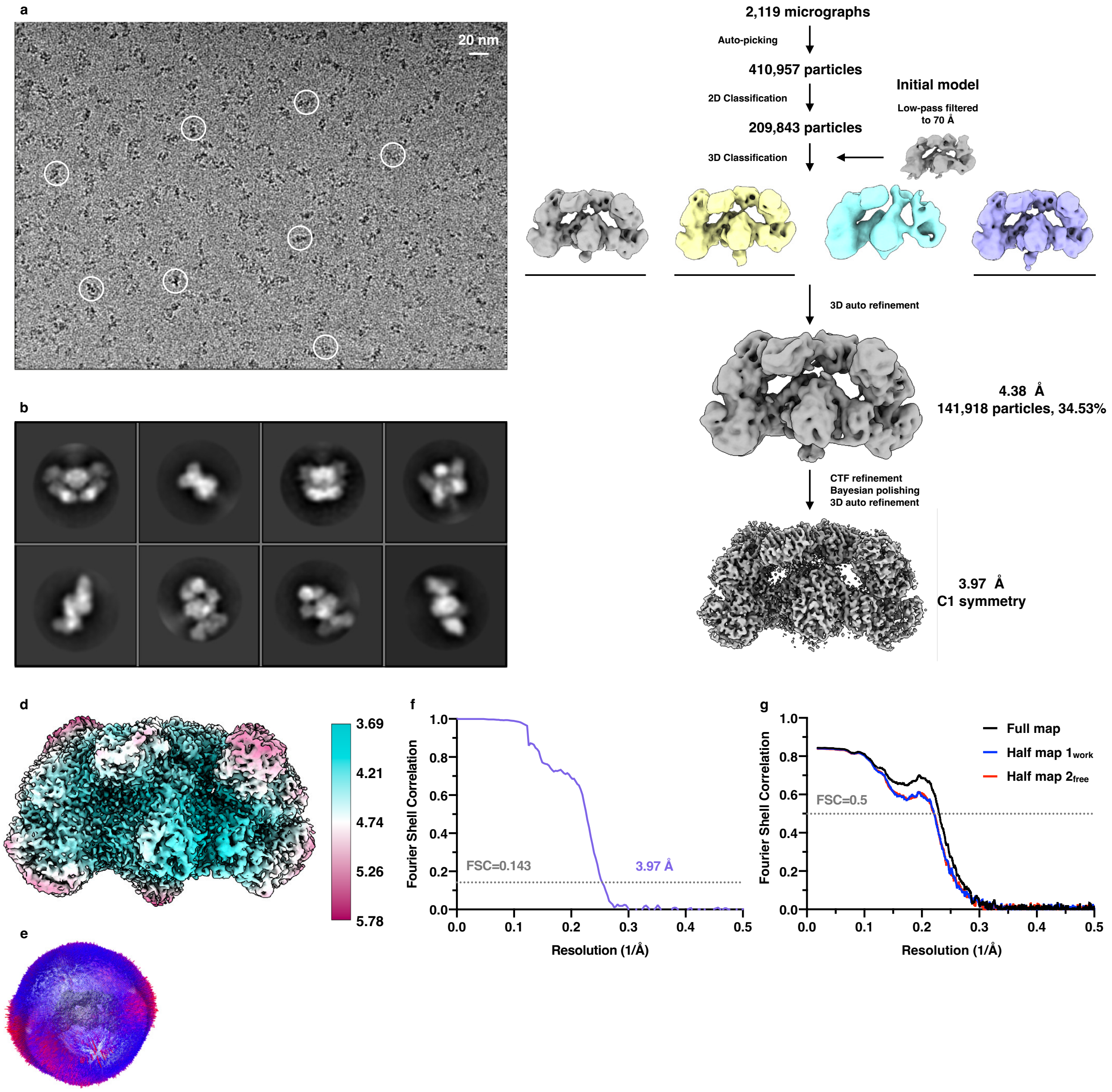

Supplementary Fig. 22 Cryo-EM structure determination of CalA3 with the hydrolysis product. a One representative cryo-EM micrograph from the 2119 movie stacks of CalA3 with selected particles in white circles. Scale bar, 20 nm. At least three independent experiments were repeated with similar results. **b** Representative 2D class averages. **c** A data processing workflow for the resolution-labeled cryo-EM maps. **d** Local resolution of the map estimated in RELION. **e** Angular distribution of all particles used for the final reconstruction of the map. **f** Gold-standard FSC curves of the resolution-labeled cryo-EM map (FSC=0.143 criterion). **g** FSC curves of the final refined model versus the

map that it was refined against (black); of the model refined in the first of the two independent maps used for the gold-standard FSC versus that same map (blue); and of the model refined in the first of the two independent maps versus the second independent map (red). The small difference between the work and free FSC curves indicates that the model did not suffer from overfitting.

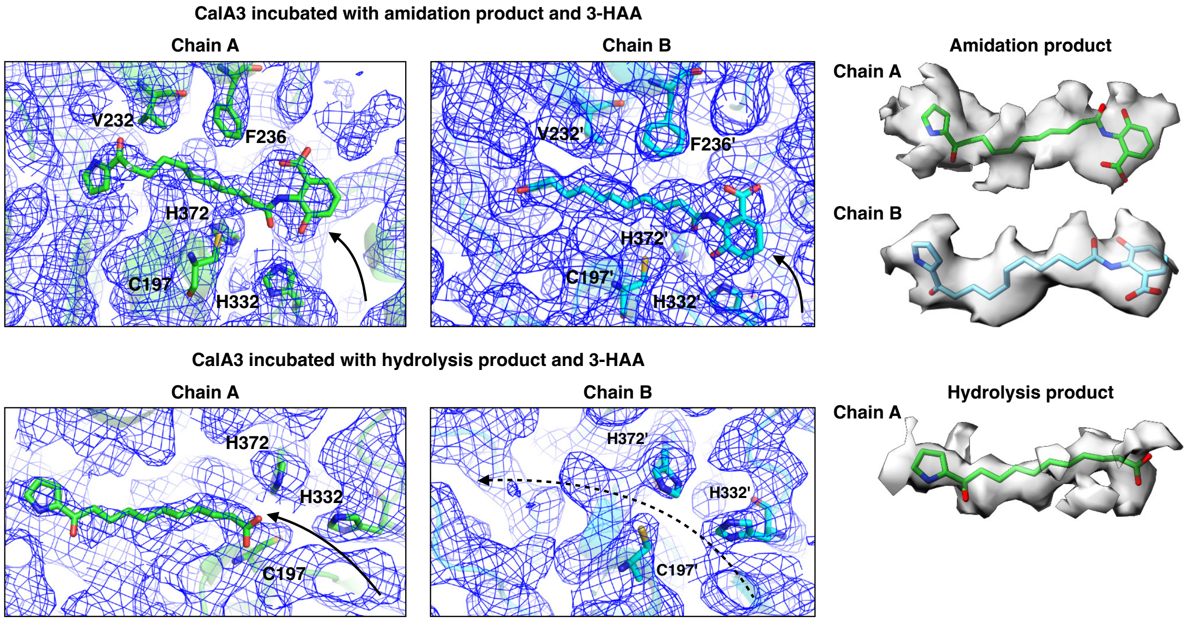

Supplementary Fig. 23 Close-up view of the substrate tunnels of CalA3 KS domains. The sharpened cryo-EM maps of the substrate tunnel regions of CalA3 are shown as mesh. The map incubated with the amidation products of chain A and B is contoured at 10.3σ and 7.9σ respectively. The map incubated with the hydrolysis product of chain A and B is contoured at 7.7σ and 10.6σ respectively. The solid arrows point at the map for each product. The dotted arrows represent the direction of substrate entry without observing the ligands. The figure was generated in Pymol. The cryo-EM maps of ligands, shown on the right as surfaces, are contoured at 5.6σ and 6.5σ for the amidation products of chain A and B respectively, and at 6.3σ for the hydrolysis product of chain A and generated in ChimeraX. The carving distance is 2.5 Å.

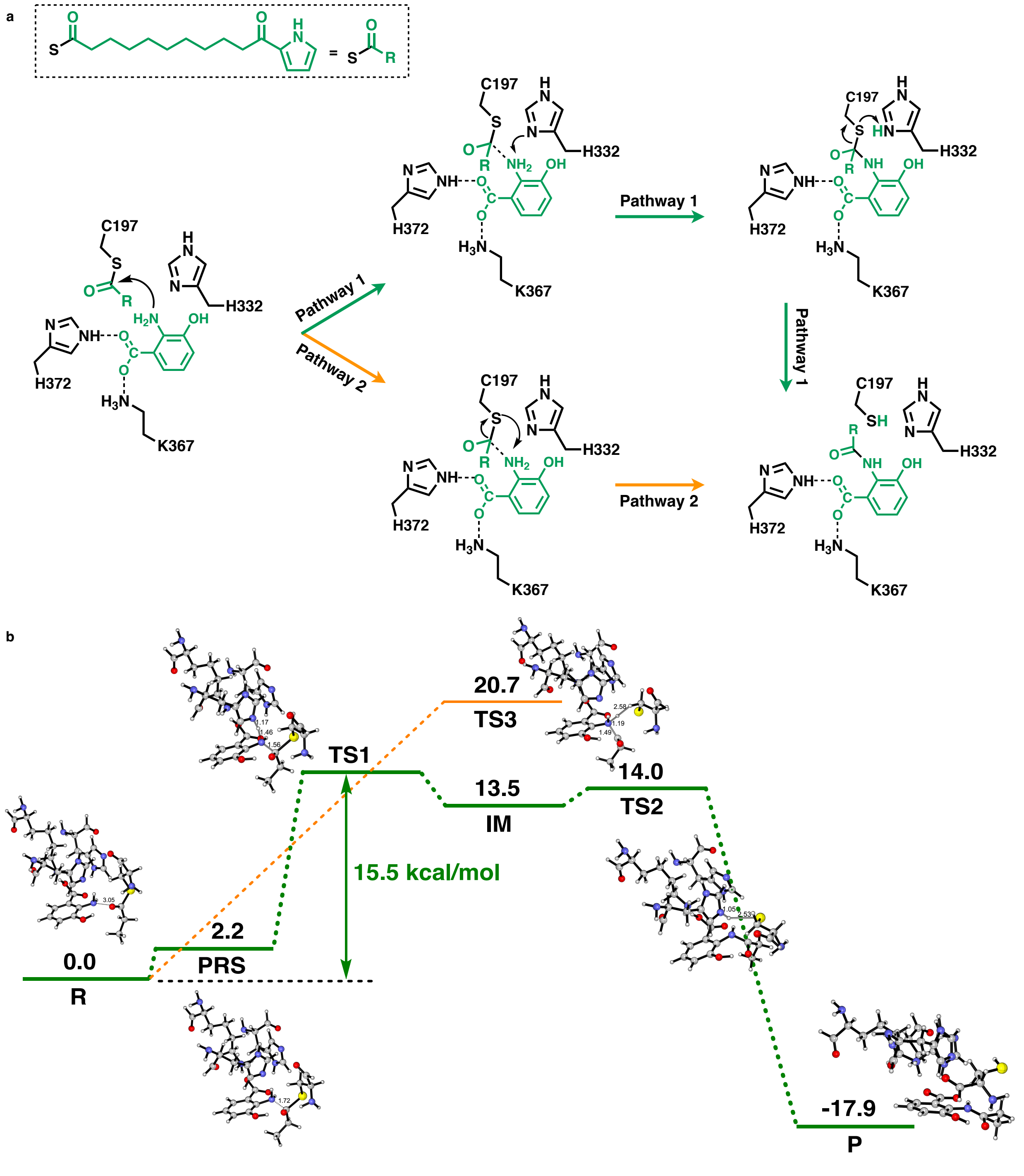

Supplementary Fig. 24 Proposed amidation mechanism of CalA3 KS domain. **a** Proposed two reaction mechanism pathways. Different from pathway 1, in pathway 2 the hydrogen of 3-HAA's amino group could be directly extracted by sulfur of C197 without the assistance of H332; at the same time, the amino group of 3-HAA attacks C1 of the acylated SNAC-N1 to form a sub-3-HAA intermediate and the refreshed C197. **b** Potential energy profile of two pathways. R, ground state; PRS, pre-reaction state; TS1, 2 and 3, transition state 1, 2 and 3; IM, intermediate state; P, product state.

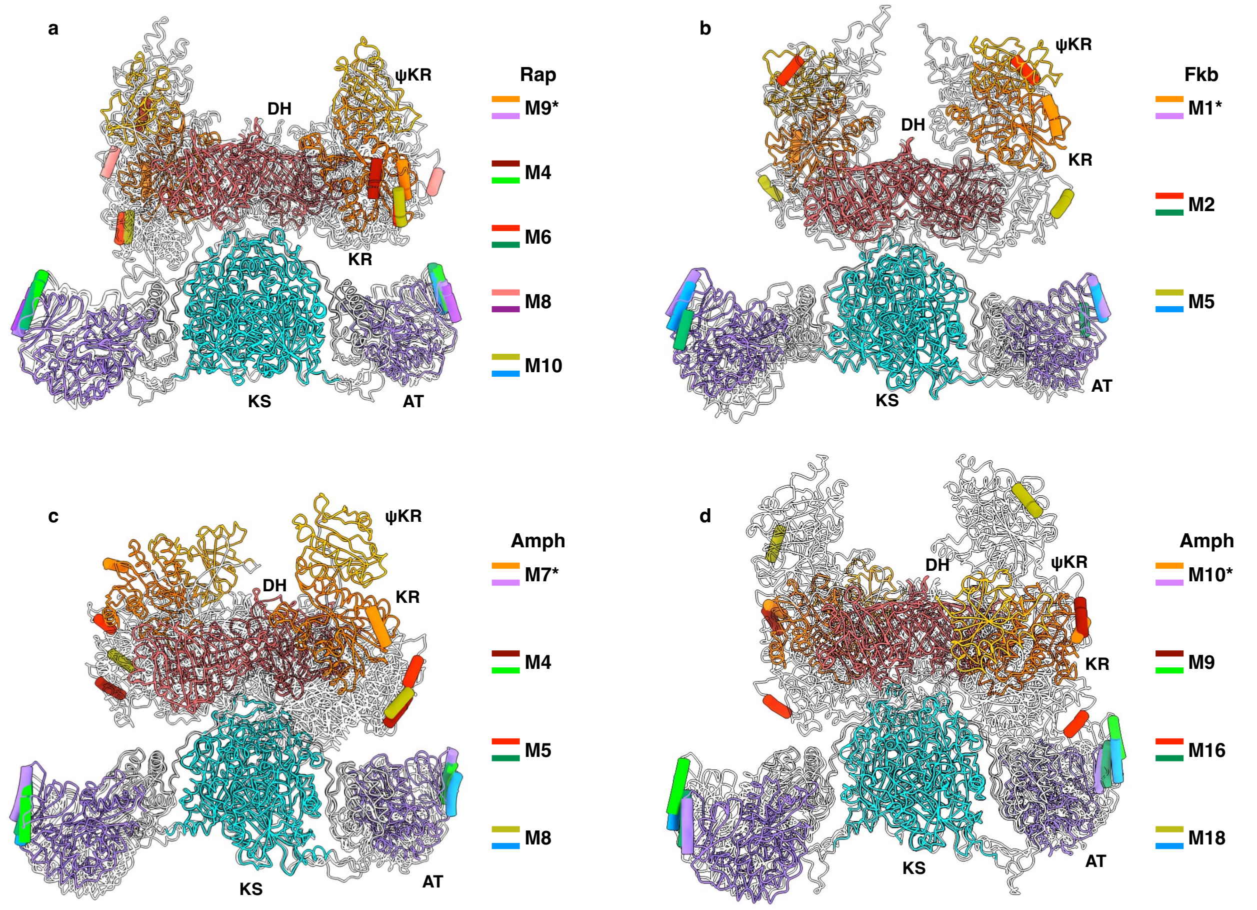

Supplementary Fig. 25 Structural prediction of KS-AT-DH-KR-ACP arranged modular PKSs. Sixteen modular PKSs are predicted by AlphaFold2, shown in line representations, and the DDs and the flexible ACP domains are omitted on purpose. These megaenzymes are involved in the biosynthesis of rapamycin, FK-506 and amphotericin B, respectively. Condensing and modifying regions of Rap M9, Fkb M1, Amph M7 and M10 are aligned respectively to the KS and DH dimers of CalA3; and each domain of the four structures are colored uniquely to reveal the architectures (marked by asterisks). The other PKSs are superposed onto these four and colored light gray, but a helix of AT and a helix of KR domain thereof are shown in tubular cartoon representation, highlighting the structural variations of condensing and modifying regions of each PKS, respectively. **a** Structures of Rap module 4, 6, 8, 9 and 10. **b** Structures of Fkb module 1, 2 and 5. **c** Structures of Amph module 4, 5, 7 and 8. **d** Structures of Amph module 9, 10, 17 and 18.

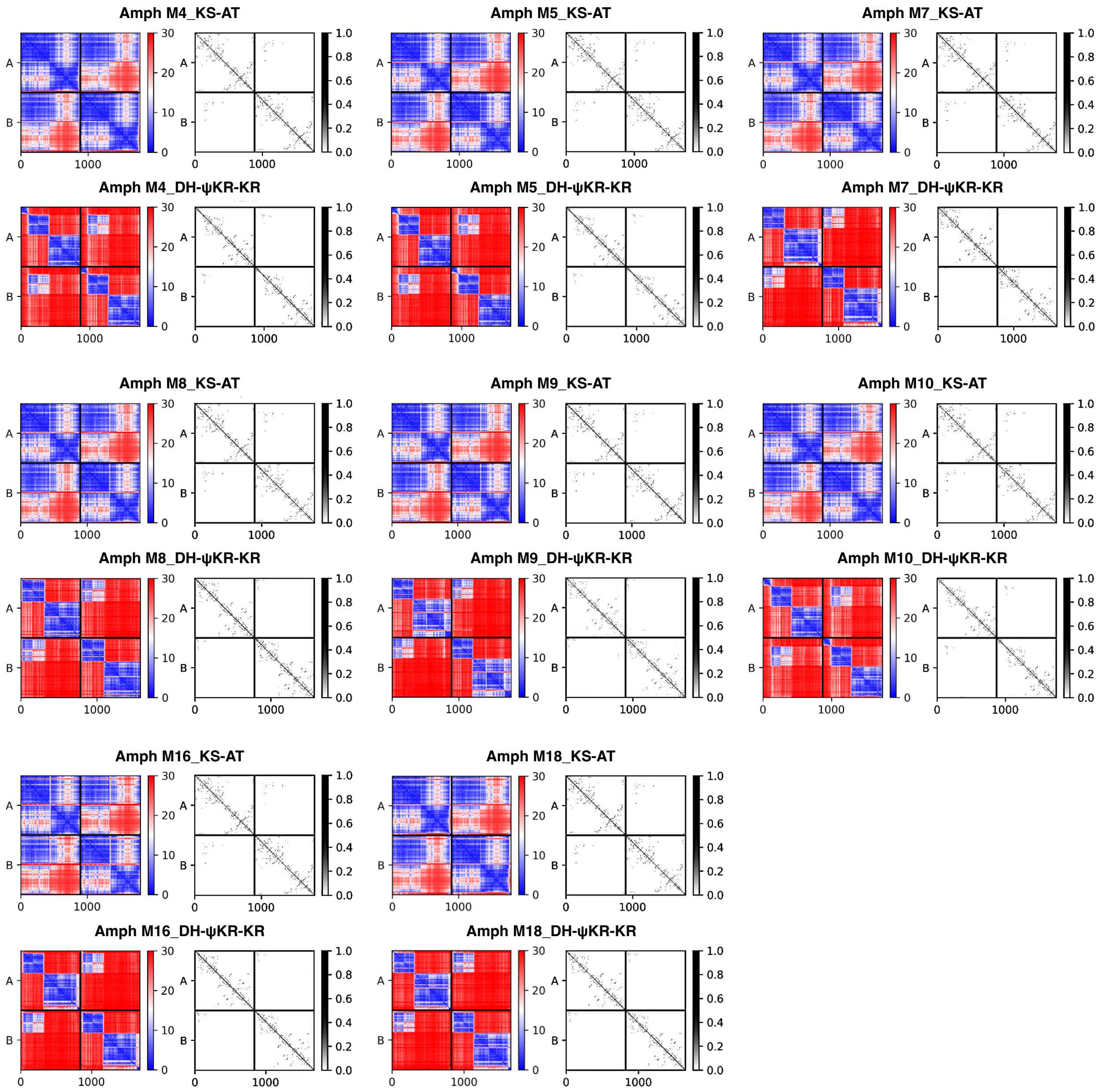

Supplementary Fig. 26 The confidence prediction of the AlphaFold2-predicted structures (part 1). Each panel of Amph M4, 5, 7, 8, 9, 10, 16 and 18 shows two plots. Left, the predicted aligned error (PAE) plot which helps to assess the reliability of relative domain positions and orientations; right, the contact probability plot which indicates the probability of the two domains interaction.

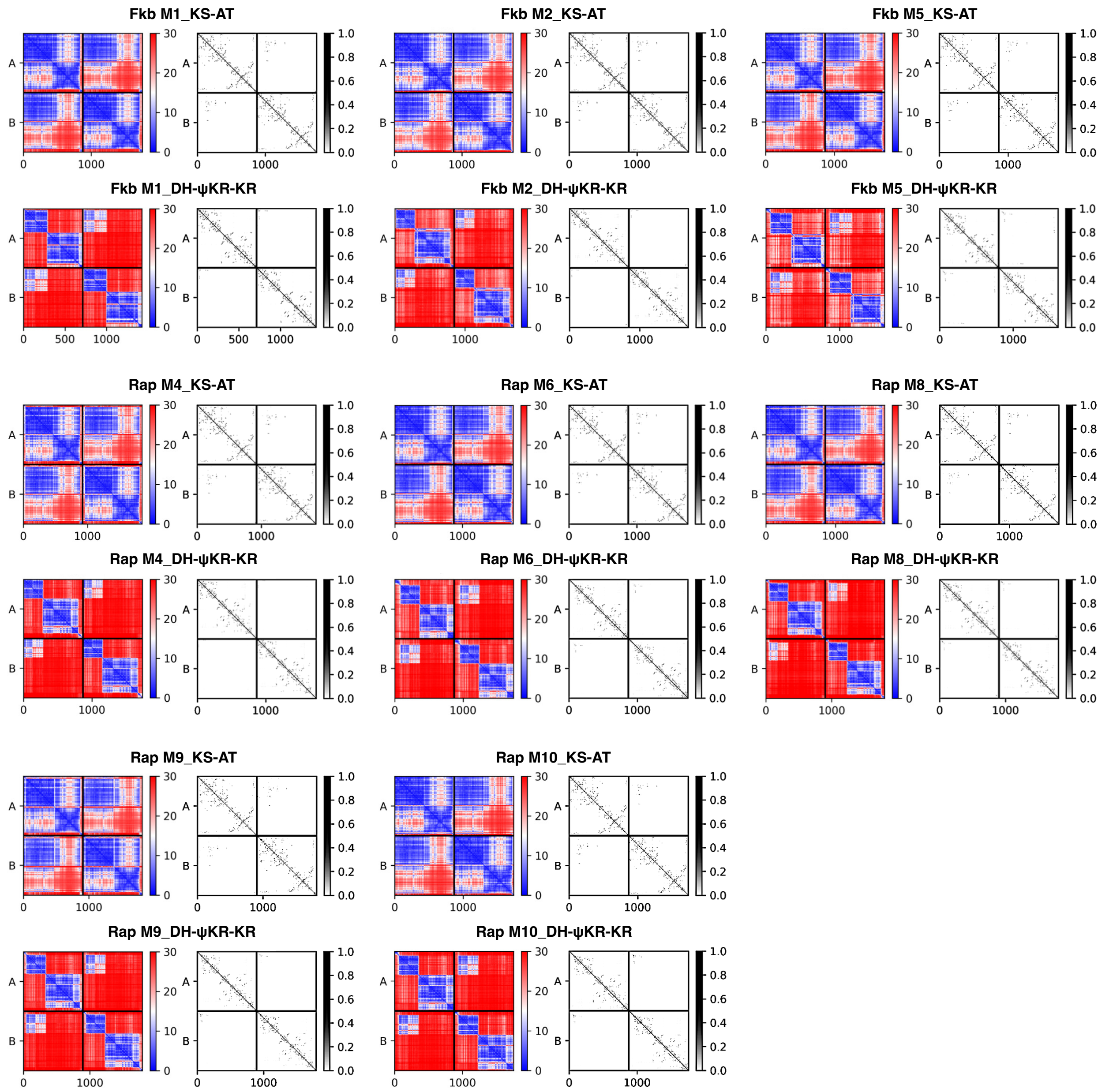

Supplementary Fig. 27 The confidence prediction of the AlphaFold2-predicted structures (part 2). Each panel of Fkb M1, M2 and M5; Rap M4, M6, M8, M9 and M10 shows two plots. Left, the predicted aligned error (PAE) plot which helps to assess the reliability of relative domain positions and orientations; right, the contact probability plot which indicates the probability of the two domains interaction.

a

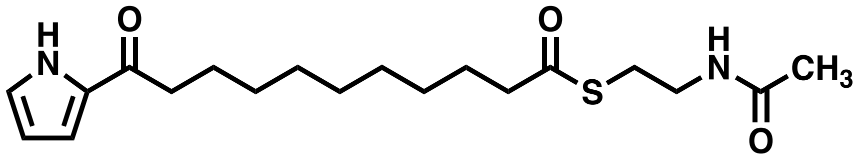

H
N
O
O
S
H
N
CH3
O

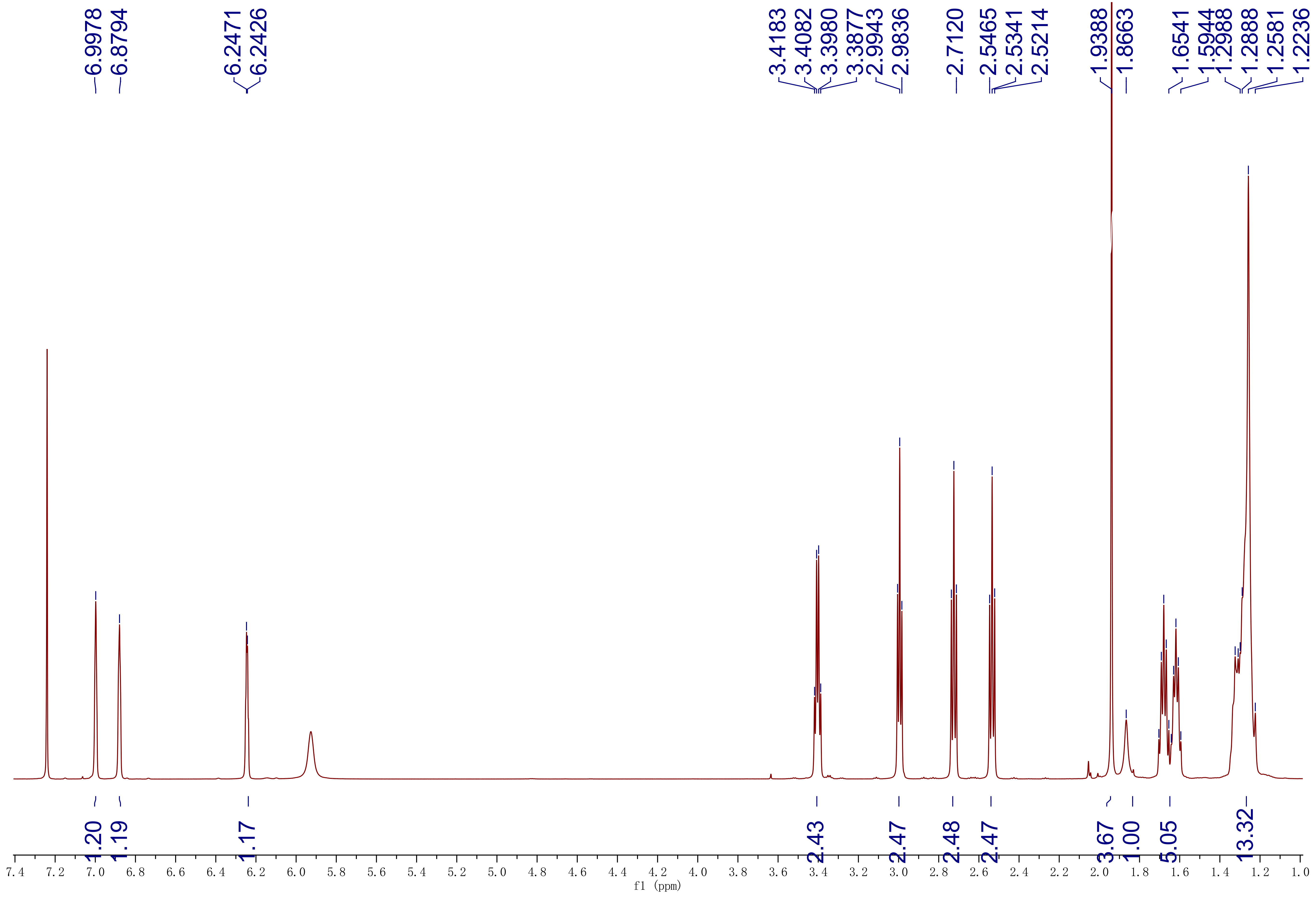

6.9978
6.8794
6.2471
6.2426
3.4183
3.4082
3.3980
3.3877
2.9943
2.9836
2.7120
2.5465
2.5341
2.5214
1.9388
1.8663
1.6541
1.5944
1.2988
1.2888
1.2581
1.2236
1.20
1.19
1.17
2.43
2.47
2.48
2.47
3.67
1.00
5.05
13.32
7.4 7.2 7.0 6.8 6.6 6.4 6.2 6.0 5.8 5.6 5.4 5.2 5.0 4.8 4.6 4.4 4.2 4.0 3.8 3.6 3.4 3.2 3.0 2.8 2.6 2.4 2.2 2.0 1.8 1.6 1.4 1.2 1.0
f1 (ppm)

b

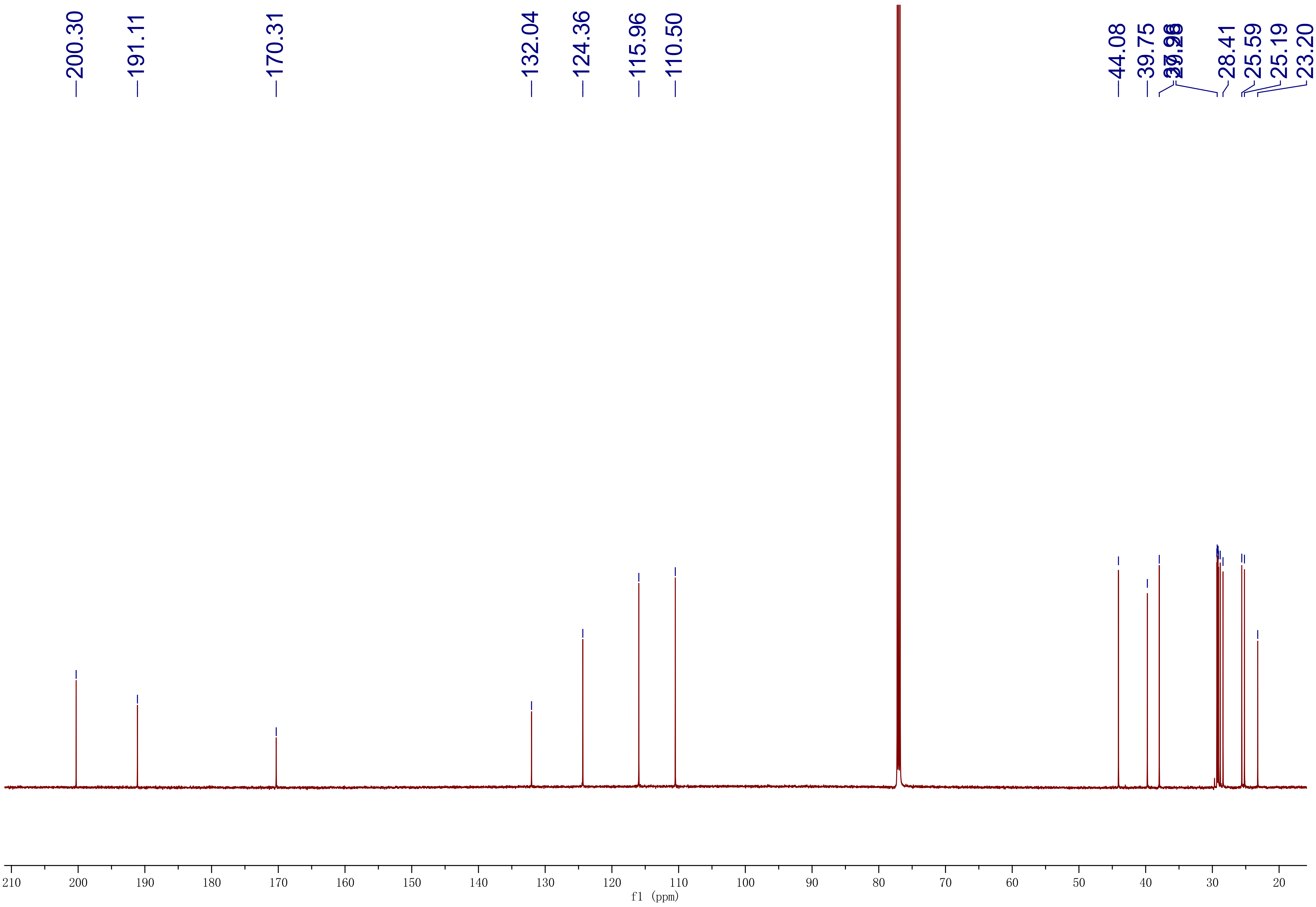
200.30
191.11
170.31
132.04
124.36
115.96
110.50
44.08
39.75
28.41
25.59
25.19
23.20
210 200 190 180 170 160 150 140 130 120 110 100 90 80 70 60 50 40 30 20
f1 (ppm)

c

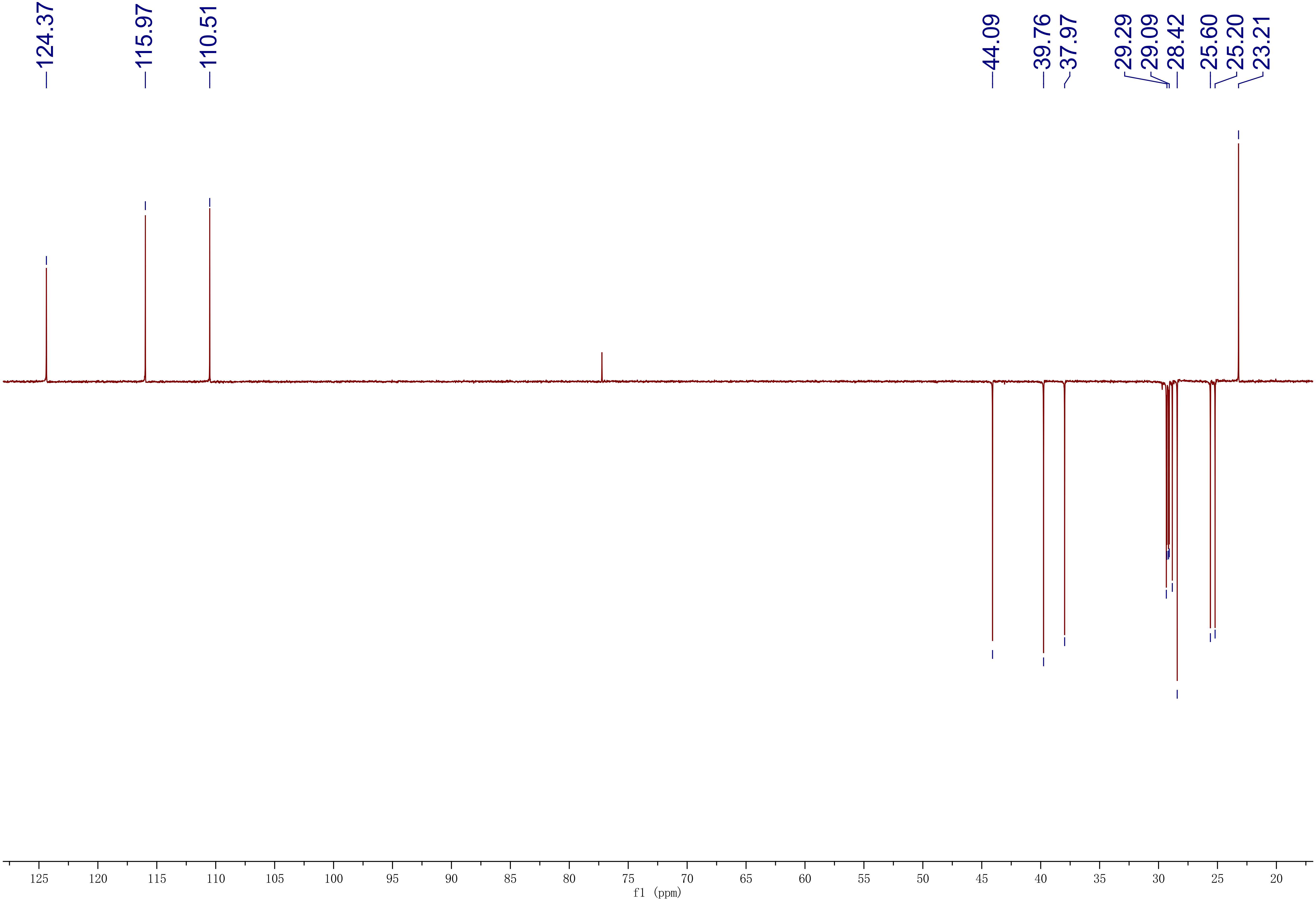
124.37
115.97
110.51
44.09
39.76
37.97
29.29
29.09
28.42
25.60
25.20
23.21
125
120
115
110
105
100
95
90
85
80
75
70
65
60
55
50
45
40
35
30
25
20
f1 (ppm)

d

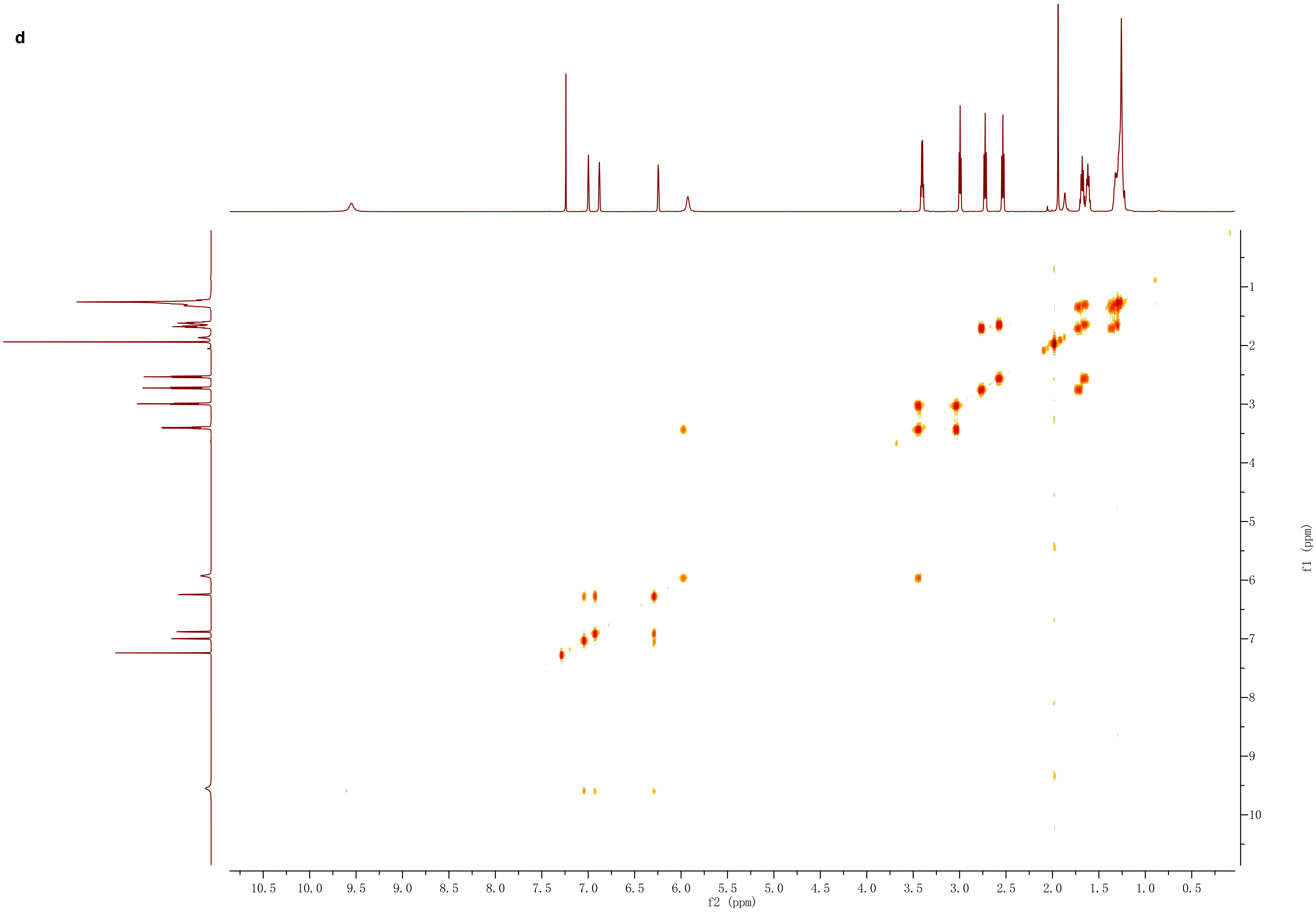

f2 (ppm)
f1 (ppm)
10.5
10.0
9.5
9.0
8.5
8.0
7.5
7.0
6.5
6.0
5.5
5.0
4.5
4.0
3.5
3.0
2.5
2.0
1.5
1.0
0.5
1
2
3
4
5
6
7
8
9
10

e

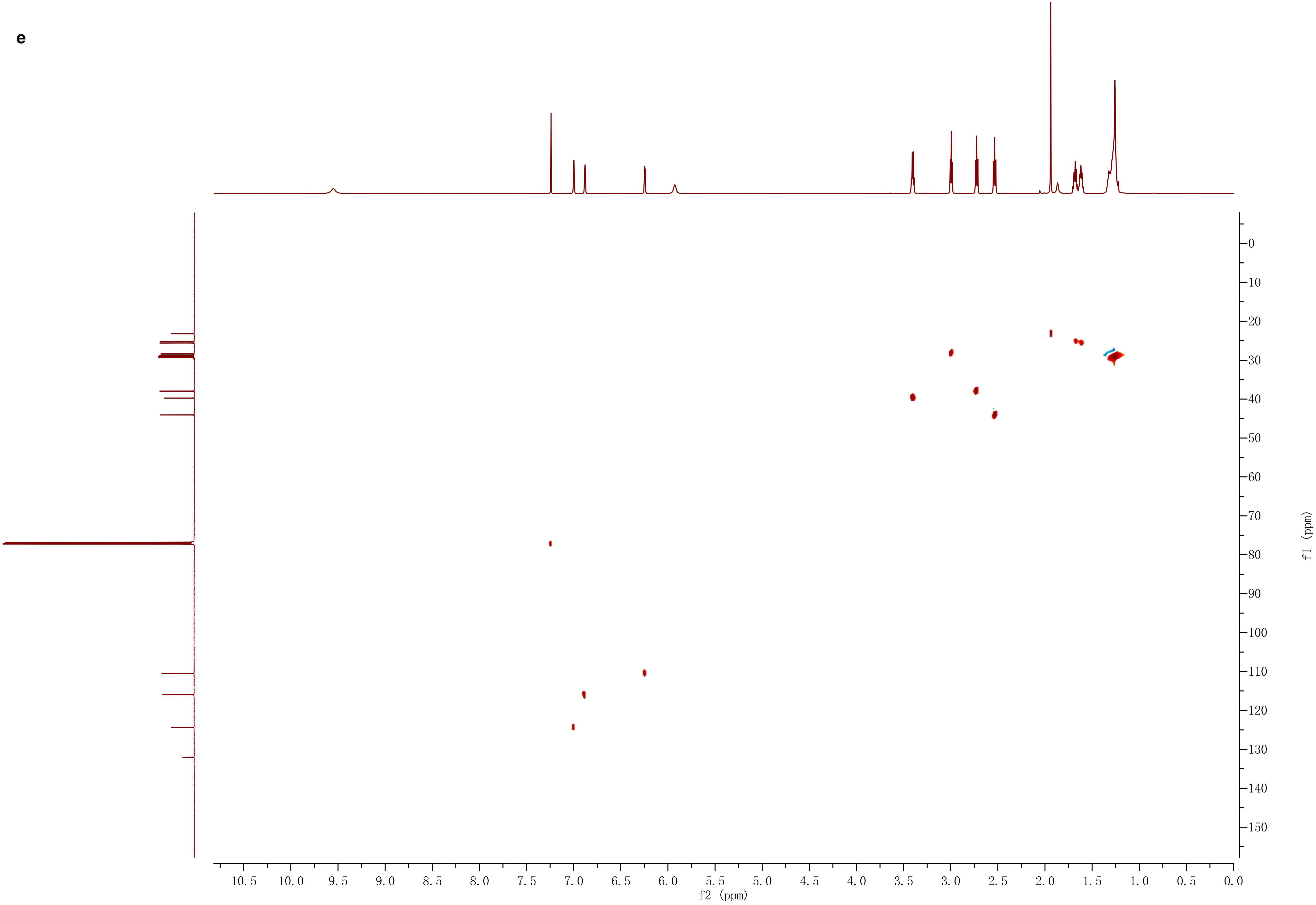

0
10
20
30
40
50
60
70
80
90
100
110
120
130
140
150
f1 (ppm)
10.5
10.0
9.5
9.0
8.5
8.0
7.5
7.0
6.5
6.0
5.5
5.0
4.5
4.0
3.5
3.0
2.5
2.0
1.5
1.0
0.5
0.0
f2 (ppm)

f

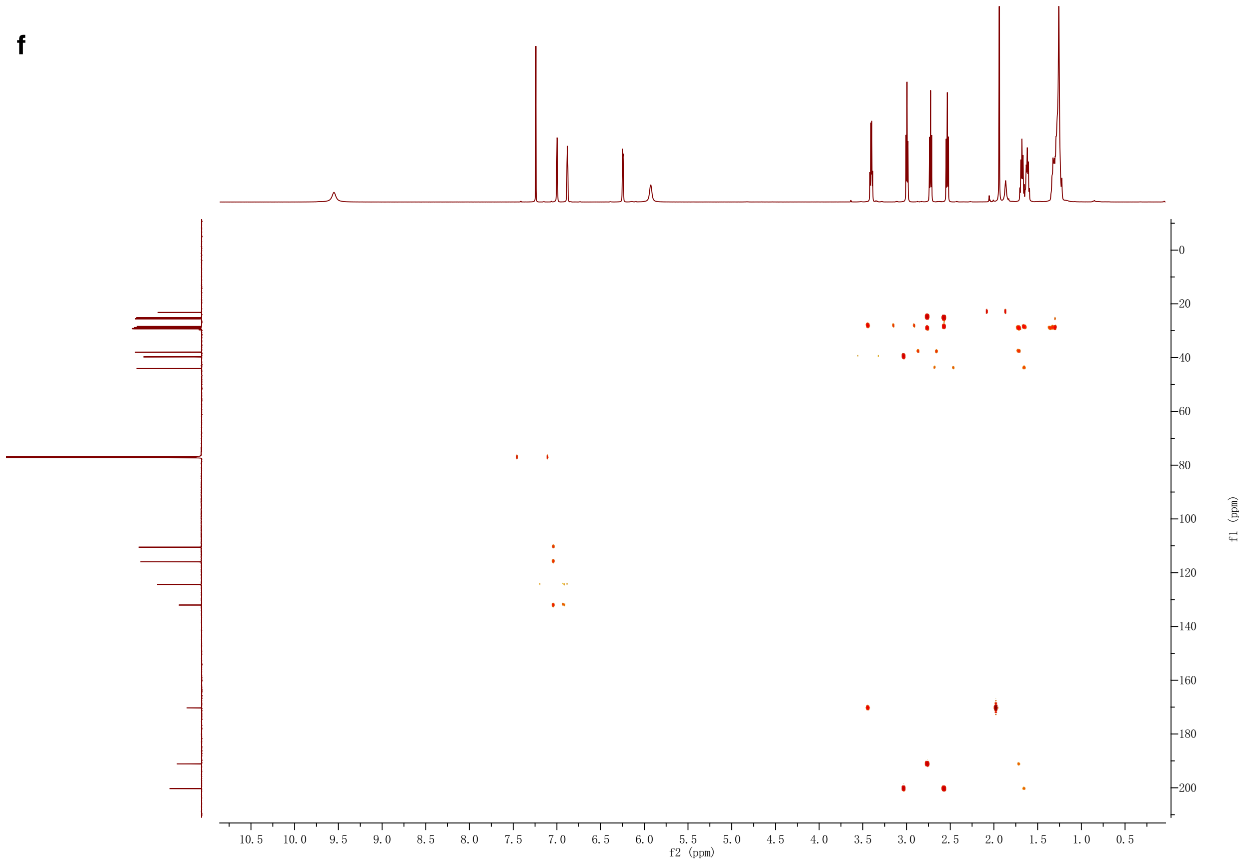

Supplementary Fig. 28 NMR spectra of SNAC-N1 (compound 1). a ^{1}H spectrum. **b** ^{13}C spectrum. **c** DEPT-135 spectrum. **d** gCOSY spectrum. **e** gHSQC spectrum. **f** gHMBC spectrum.

a

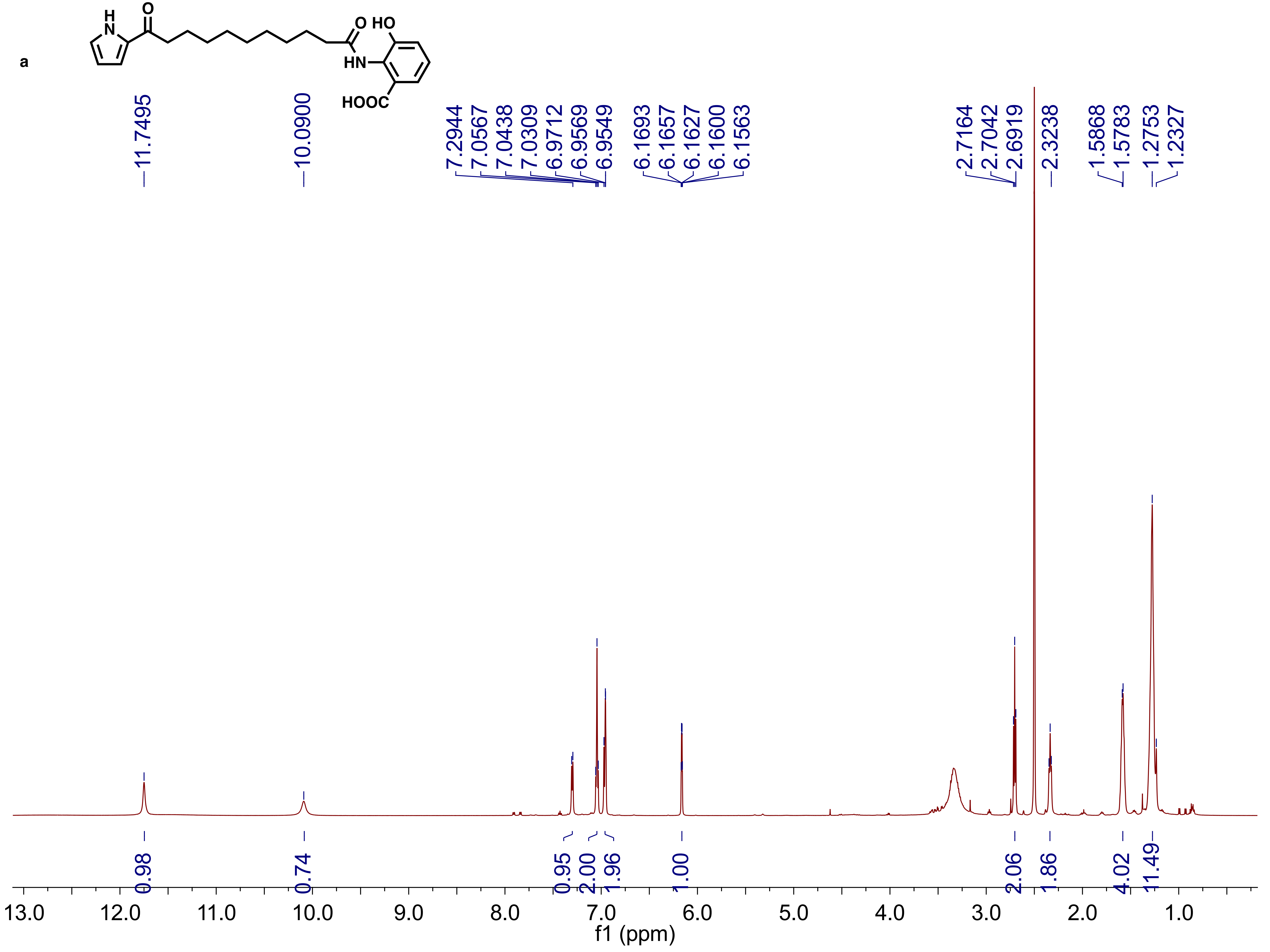
H
N
O
O HO
HN
HOOC
11.7495
10.0900
7.2944
7.0567
7.0438
7.0309
6.9712
6.9569
6.9549
6.1693
6.1657
6.1627
6.1600
6.1563
2.7164
2.7042
2.6919
2.3238
1.5868
1.5783
1.2753
1.2327
0.98
0.74
0.95
2.00
1.96
1.00
2.06
1.86
4.02
11.49
13.0
12.0
11.0
10.0
9.0
8.0
7.0
6.0
5.0
4.0
3.0
2.0
1.0
f1 (ppm)

b

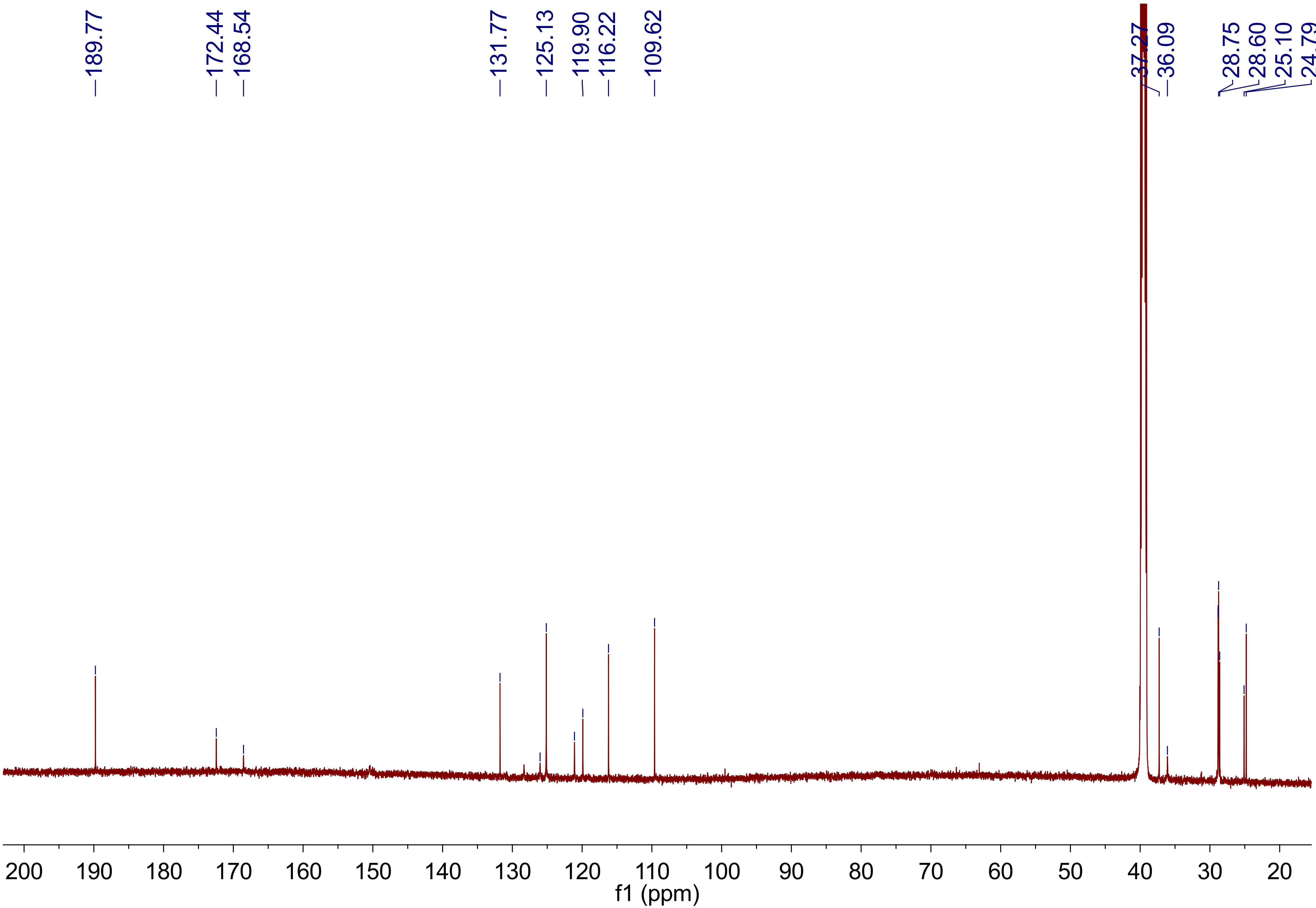

189.77
172.44
168.54
131.77
125.13
119.90
116.22
109.62
37.27
36.09
28.75
28.60
25.10
24.79
200 190 180 170 160 150 140 130 120 110 100 90 80 70 60 50 40 30 20
f1 (ppm)

c

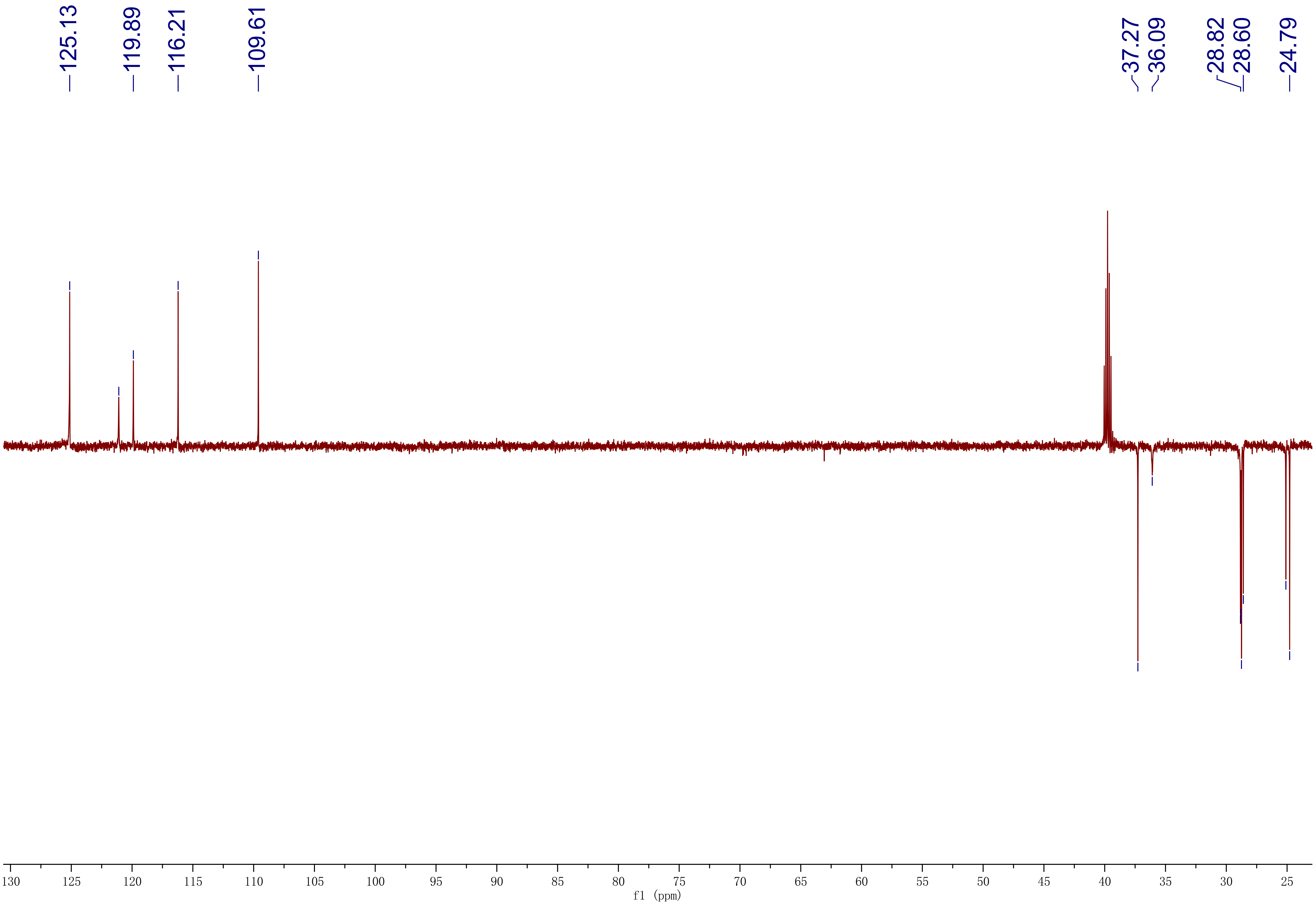

125.13
119.89
116.21
109.61
37.27
36.09
28.82
28.60
24.79
130
125
120
115
110
105
100
95
90
85
80
75
70
65
60
55
50
45
40
35
30
25
f1 (ppm)

d

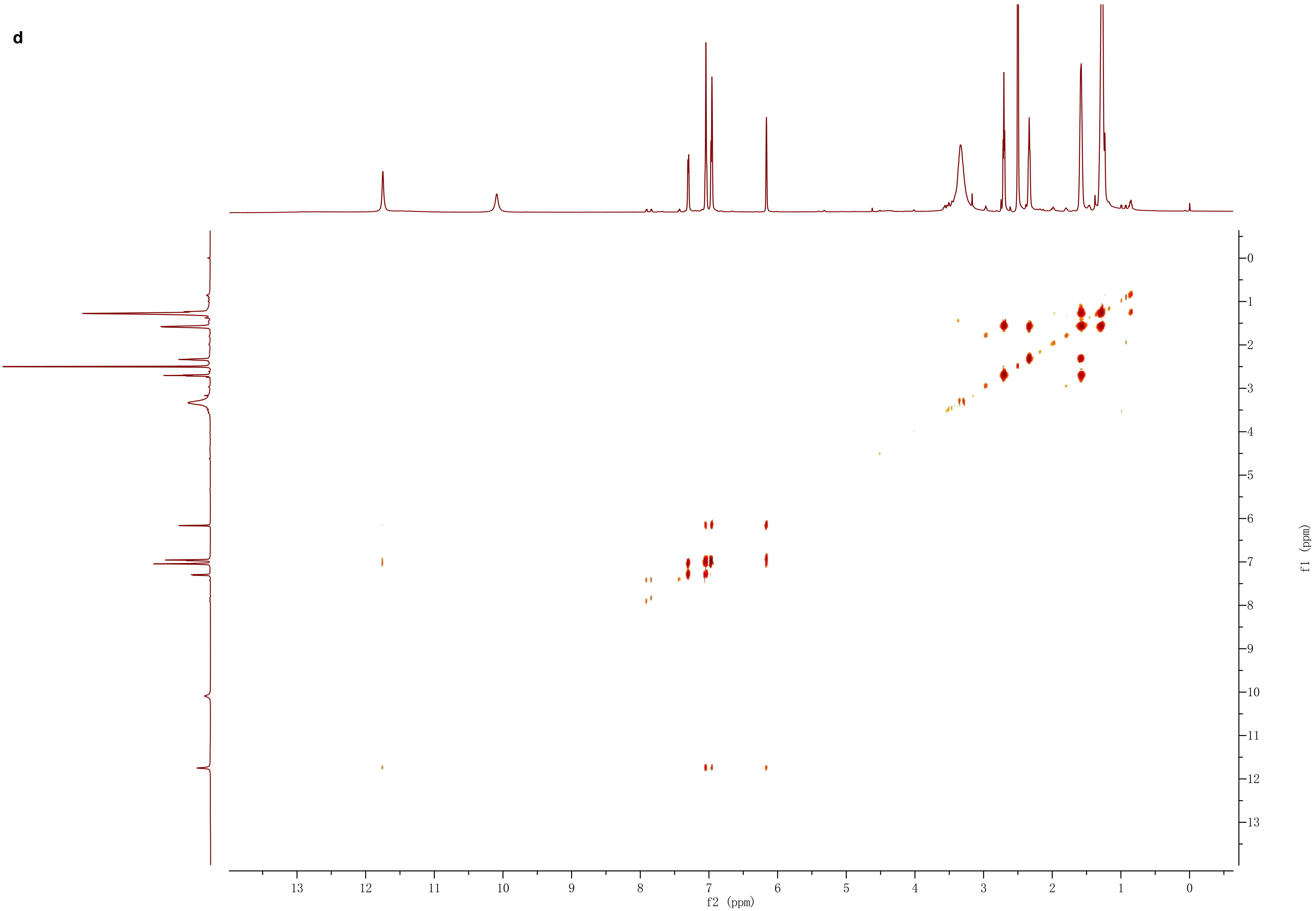

e

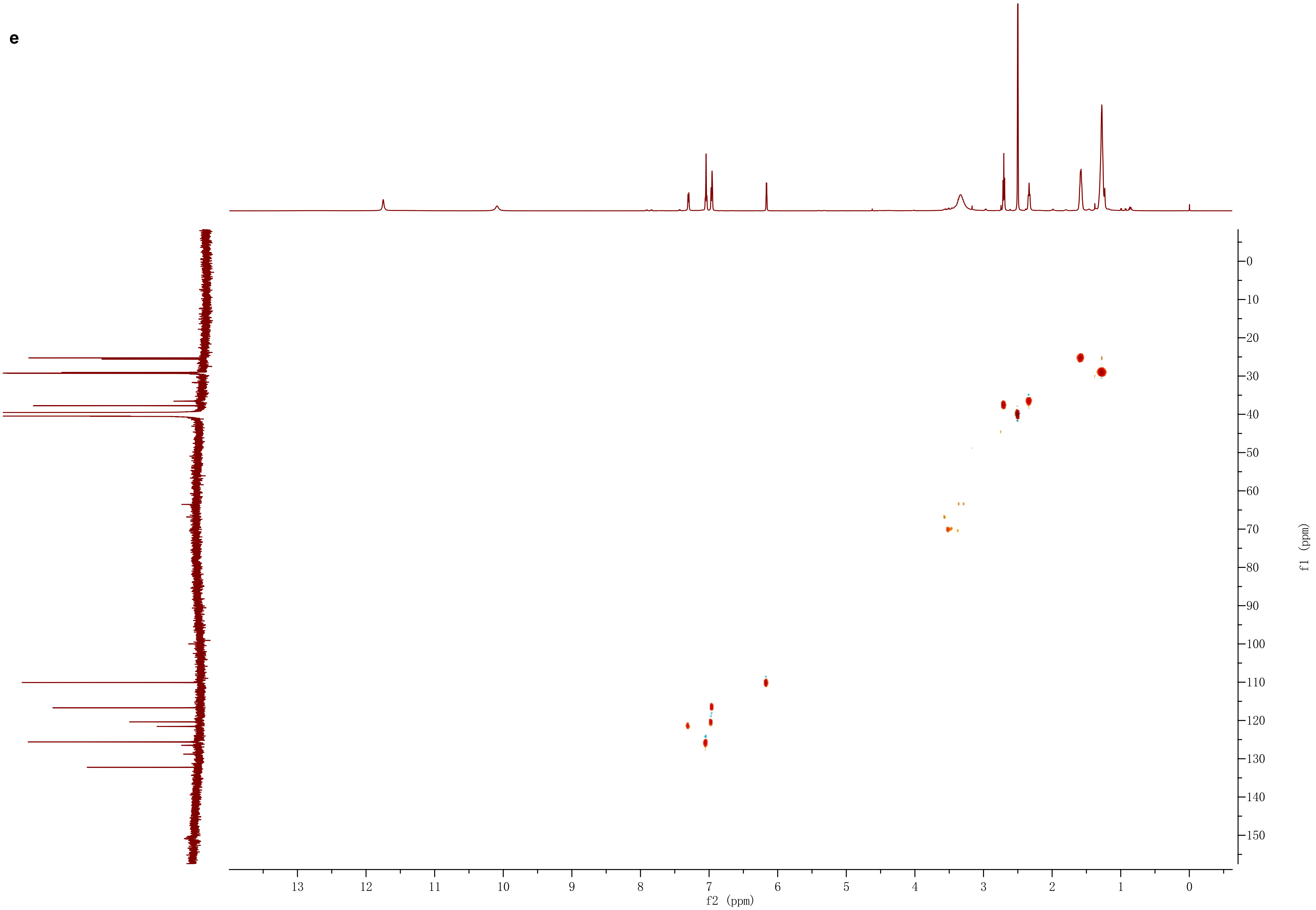

f2 (ppm)
f1 (ppm)
13
12
11
10
9
8
7
6
5
4
3
2
1
0
10
20
30
40
50
60
70
80
90
100
110
120
130
140
150

f

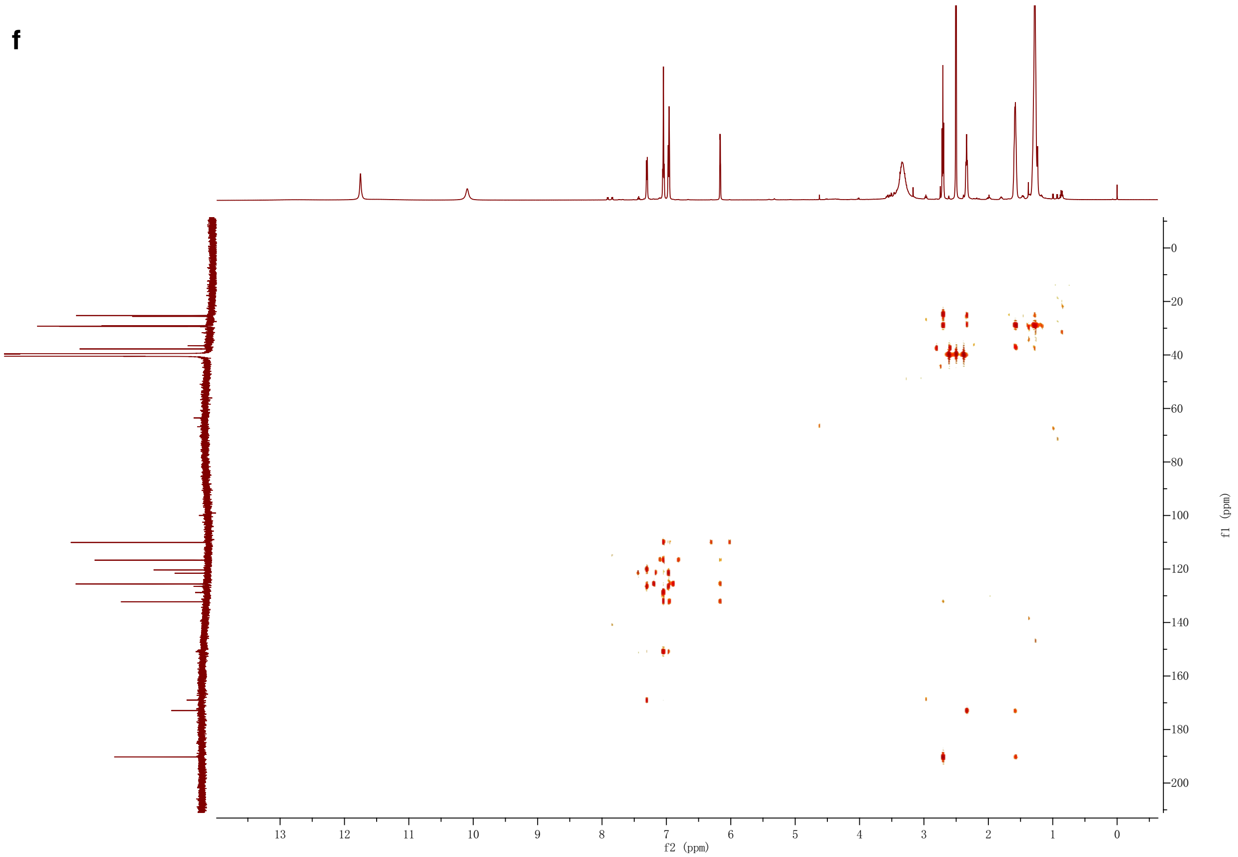

Supplementary Fig. 29 NMR spectra of amidation product (compound 3). a ^{1}H spectrum. **b** ^{13}C spectrum. **c** DEPT-135 spectrum. **d** gCOSY spectrum. **e** gHSQC spectrum. **f** gHMBC spectrum.

Supplementary Table 1. Bacterial strains and plasmids used in this study.

Strain or plasmid	Description	Reference or source
Streptomyces chartreusis NRRL 3882	Calcimycin (A23187) production, wild type	NRRL
GLX13 (*ΔcalA3*)	*calA3* deletion mutant, no calcimycin production	This work
GLX14 (*ΔcalA3::calA3*)	*calA3* complementation strain, restores calcimycin production	This work
GLX15 (*ΔcalA3::calA3_KS*)	Complementation strain of the *calA3-KS* gene fragment of *Streptomyces chartreusis* NRRL 3882 disrupted *calA3* gene mutant	This work
Escherichia coli strains		
DH10B	*recA* lacZΔM15	Invitrogen
ET12567 (pUZ8002)	Cml, Kan, *dam dcm* hsdS Tra^+ Cml	[26]
BW25113/pIJ773	K-12 derivative: *ΔaraBAD*, *ΔrhaBAD*/pBluescript KS (+), *aac* (3) IV, oriT (RK2), FRT sites	[26]
BW25113/pKD46	F-, *Δ(araD-araB)567*, *ΔlacZ4787* (::rrnB-3), λ^-, *rph-1*, *Δ(rhaD-rhaB)568*, *hsdR514*, pKD46	[26]
BAP1	F^-*omp*T *hsd*S_B (r_B^-, m_B^-) *gal dcm* (DE3) (BL21 (DE3) with sfp)	Stratagene
Plasmids		
p16F9	Cml	[27]
pJTU2170	Integrative vector for gene complementation, *aac* (3) IV from pIB139 was replaced by *bla* and *neo* cassette	[28]
pJTU3767	p16F9-derived plasmid carrying an apramycin resistance gene and a defective *calA3*	This work
pJTU3781	pJTU2170-derived plasmid carrying *calA3* for expression in *Streptomyces*	This work
pJTU3782	pJTU2170-derived plasmid carrying *calA3-KS* for expression in *Streptomyces chartreusis* (PCR amplification fragment of *calA3-KS* (1356 bp)), NdeI and EcoRI were introduced into the two ends of the PCR fragment, which was connected to the corresponding sites of pJTU2170 and used to complement the *calA3*-deletion mutant	This work
pET28CalA3_cH	pET28a (+)-derived plasmid for *calA3* expression with C-Terminal-6 His Tag, Kan^r	This work
pET28CalA3cH_C197A	Derived from pET28CalA3_cH, site mutant with C197A on CalA3, Kan^r	This work
pET28CalA3cH_H332A	Derived from pET28CalA3_cH, site mutant with H332A on CalA3, Kan^r	This work
pET28CalA3cH_H372A	Derived from pET28CalA3_cH, site mutant with H372A on CalA3, Kan^r	This work
pET28CalA3cH_K367A	Derived from pET28CalA3_cH, site mutant with H367A on CalA3, Kan^r	This work
pET28CalA3cH_L437A	Derived from pET28CalA3_cH, site mutant with L437A on CalA3, Kan^r	This work

Supplementary Table 2. Primers used in this study.

Name	Sequence
calA3-F1	CTGGACGGCGTCAACGCCGGTTTCATCCACGGCGTCGAGA TTCCGGGGATCCGTCGACC
calA3-F2	CCGTTCGTTGCGCAGCGCCATCACCAGCTTCACGATGCCT GTAGGCTGGAGCTGCTTC
calA3-F3	GGTGACGCGGTCGGCGGCCT
calA3-F4	CCCGTCGACGTGCACGGTGC
calA3-F5	GGAATTCCATATGGACCCGCAGCAGCG
calA3-F6	CCGGAATTCCCCACCTCGACATCGAGAAGT
f28calA3cH-S	**ATATACCATGCCCGACGA**AGAGAAACTGCAGAAG
f28calA3cH-A	**GGCCGCGCACCCTTC**TTCTTACGGGCCCCCTT
v28calA3cH-S	**AAGAAGGGTGCGGCC**GCACTCGAGCACCA
v28calA3cH-A	**TCGTCGGGCATGGTATAT**CTCCTTCTTAAAG
cCalA3-C197A-S	CCAG**GCG**ACCTCCTCGATCGTGGCCC
cCalA3-C197A-A	GGT**CGC**CTGGGTGTCGACGGTGACC
cCalA3-H332A-S	GTGGGTGCGACGCTGGGCGACGGCGTCGAGGTGAC
cCalA3-H332A-A	TCGCACCCACACC**CGC**CCCCTCCACCGCGTCGACG
cCalA3-H372A-S	AAGCAATATCGGT**GCC**ACCCAGACGGTGGGCGCC
cCalA3-H372A-A	GATATTGCTTTTCACCGAGCCCAGCAGCAACGGCC
cCalA3-K367A-S	CGTC**GC**GTCCAACATCGGCCACACCC
cCalA3-K367A-A	GGAC**GC**GACGGAGCCCAGCAGCAAC
cCalA3-L437A-S	**GCG**ACCCTGAGCGGCACGAACGGCCATC
cCalA3-L437A-A	TCAGGGT**CGC**ACAGGTCAGACCGGCGCGC

Supplementary Table 3. Data analysis of the ^{1}H-NMR and ^{13}C-NMR spectra of SNAC-N1 (compound 1).

No.	δ_H (mult, *J*/Hz)[a]	δ_C (DEPT)[b]	^{1}H-^{1}H COSY	gHMBC
1	7.00 (brs)	124.4 (d)	2	4, 3
2	6.24 (brs)	110.5 (d)	1, 3	4
3	6.87 (brs)	116.0 (d)	2	5
4		132.0 (s)		
5		191.2 (s)		
6	2.85 (dd, 7.38, 7.44)	38.0 (t)	7	4, 8
7	1.69 (m)	25.2 (t)	6, 8	5, 9
8	1.30 (m)	29.1 (t)	7, 9	6, 10
9	1.30 (m)	29.3 (t)	8, 10	7, 11
10	1.30 (m)	29.3 (t)	9, 11	8, 12
11	1.30 (m)	29.2 (t)	10, 12	9, 13
12	1.30 (m)	28.8 (t)	11, 13	10, 14
13	1.62 (m)	25.6 (t)	12, 14	11, 15
14	2.53 (dd, 7.44, 7.62)	44.0 (t)	13	12
15		200.3 (s)		
17	2.99 (t, 6.42)	28.4 (t)	18	15
18	3.38 (m)	39.8 (t)	17	20
20		170.3 (s)		
21	1.94 (s)	23.2 (q)		20

Supplementary Table 4. Data analysis of the ^{1}H-NMR and ^{13}C-NMR spectra of amidation product (compound 3).

No.	δ_H (mult, *J*/Hz)[a]	δ_C (DEPT)[b]	^{1}H-^{1}H COSY	gHMBC
1	7.00 (brs)	124.4 (d)	2	4, 3
2	6.24 (brs)	110.5 (d)	1, 3	4
3	6.87 (brs)	116.0 (d)	2	5
4		132.0 (s)		
5		191.2 (s)		
6	2.85 (dd, 7.38, 7.44)	38.0 (t)	7	4, 8
7	1.69 (m)	25.2 (t)	6, 8	5, 9
8	1.30 (m)	29.1 (t)	7, 9	6, 10
9	1.30 (m)	29.3 (t)	8, 10	7, 11
10	1.30 (m)	29.3 (t)	9, 11	8, 12
11	1.30 (m)	29.2 (t)	10, 12	9, 13
12	1.30 (m)	28.8 (t)	11, 13	10, 14
13	1.62 (m)	25.6 (t)	12, 14	11, 15
14	2.53 (dd, 7.44, 7.62)	44.0 (t)	13	12
15		200.3 (s)		
17	2.99 (t, 6.42)	28.4 (t)	18	15
18	3.38 (m)	39.8 (t)	17	20
20		170.3 (s)		
21	1.94 (s)	23.2 (q)		20

Supplementary Table 5. Cryo-EM data collection, processing and validation statistics.

	Flanked docking domain	Perpendicular docking domain
	EMD-32863<br>PDB 7WVZ	EMD-32864
**Data collection and processing**		
Magnification	64000	64000
Voltage (kV)	300	300
Electron exposure (e-/Å^2)	48.6	48.6
Defocus range (μm)	-1.5 to -2.5	-1.5 to -2.5
Pixel size (Å)	1.10	1.10
Symmetry imposed	C1	C1
Final particle images (no.)	224,991	36,984
Map resolution (Å)	3.38	4.55
FSC threshold	0.143	0.143
Map resolution range (Å)	3.19-10.18	4.19-9.53
Map sharpening B-factor (Å)	-114.378	-143.934
**Model composition**		
Chains	2	
Non-hydrogen atoms	25,065	
Protein residues	3,430	
Ligand	0	
**R.m.s. deviations**		
Bond lengths (Å)	0.011	
Bond angles (°)	1.890	
**Validation**		
MolProbity score	1.00	
Clashscore	0.20	
Favored rotamers (%)	95.62	
Poor rotamers (%)	1.38	
**Ramachandran plot**		
Favored (%)	95.50	
Allowed (%)	4.35	
Disallowed (%)	0.15	

Supplementary Table 6. Cryo-EM data collection, processing and validation statistics.

	CalA3 with amidation product	CalA3 with hydrolysis product
	EMD-35188<br>PDB 8I4Y	EMD-35189<br>PDB 8I4Z
**Data collection and processing**		
Magnification	81000	81000
Voltage (kV)	300	300
Electron exposure (e-/Å^2)	48.06	48.06
Defocus range (μm)	-1.0 to -2.0	-1.0 to -2.0
Pixel size (Å)	0.89	0.89
Symmetry imposed	C1	C1
Final particle images (no.)	223,652	141,918
Map resolution (Å)	3.84	3.97
FSC threshold	0.143	0.143
Map resolution range (Å)	3.57-8.48	3.69-5.78
Map sharpening B-factor (Å)	-118.526	-121.562
**Model composition**		
Chains	2	2
Non-hydrogen atoms	24,610	24,610
Protein residues	3,374	3,374
Ligand	2	1
**R.m.s. deviations**		
Bond lengths (Å)	0.012	0.012
Bond angles (°)	1.940	1.923
**Validation**		
MolProbity score	1.00	0.94
Clashscore	0.55	0.53
Favored rotamers (%)	95.87	96.53
Poor rotamers (%)	1.16	0.74
**Ramachandran plot**		
Favored (%)	96.11	96.17
Allowed (%)	3.86	3.80
Disallowed (%)	0.03	0.03

Supplementary References

1. Gou, L. et al. Mutasynthesis of pyrrole spiroketal compound using calcimycin 3-hydroxy anthranilic acid biosynthetic mutant. *Appl. Microbiol. Biotechnol.* **97**, 8183–8191 (2013).

2. Sanchez-Garcia, R. et al. DeepEMhancer: a deep learning solution for cryo-EM volume post-processing. *Commun. Biol.* **4**, 874 (2021).

3. Tang, Y., Chen, A. Y., Kim, C.-Y., Cane, D. E. & Khosla, C. Structural and mechanistic analysis of protein interactions in module 3 of the 6-deoxyerythronolide B synthase. *Chem. Biol.* **14**, 931–943 (2007).
4. Tang, Y., Kim, C.-Y., Mathews, I. I., Cane, D. E. & Khosla, C. The 2.7-Angstrom crystal structure of a 194-kDa homodimeric fragment of the 6-deoxyerythronolide B synthase. *Proc. Natl. Acad. Sci. U.S.A* **103**, 11124–11129 (2006).
5. Whicher, J. R. et al. Cyanobacterial polyketide synthase docking domains: a tool for engineering natural product biosynthesis. *Chem. Biol.* **20**, 1340–1351 (2013).
6. Bagde, S. R., Mathews, I. I., Fromme, J. C. & Kim, C.-Y. Modular polyketide synthase contains two reaction chambers that operate asynchronously. *Science* **374**, 723–729 (2021).
7. Herbst, D. A., Jakob, R. P., Zähringer, F. & Maier, T. Mycocerosic acid synthase exemplifies the architecture of reducing polyketide synthases. *Nature* **531**, 533–537 (2016).
8. Pappenberger, G. et al. Structure of the human fatty acid synthase KS–MAT didomain as a framework for inhibitor design. *J. Mol. Biol.* **397**, 508–519 (2010).
9. Maier, T., Leibundgut, M. & Ban, N. The crystal structure of a mammalian fatty acid synthase. *Science* **321**, 1315–1322 (2008).
10. Wang, J. et al. Structural basis for the biosynthesis of lovastatin. *Nat. Commun.* **12**, 867 (2021).
11. Keatinge-Clay, A. Crystal structure of the erythromycin polyketide synthase dehydratase. *J. Mol. Biol.* **384**, 941–953 (2008).
12. Gay, D., You, Y.-O., Keatinge-Clay, A. & Cane, D. E. Structure and stereospecificity of the dehydratase domain from the terminal module of the rifamycin polyketide synthase. *Biochemistry* **52**, 8916–8928 (2013).
13. Akey, D. L. et al. Crystal structures of dehydratase domains from the curacin polyketide biosynthetic pathway. *Structure* **18**, 94–105 (2010).
14. Faille, A. et al. Insights into substrate modification by dehydratases from type I polyketide synthases. *J. Mol. Biol.* **429**, 1554–1569 (2017).
15. Keatinge-Clay, A. T. et al. Catalysis, specificity, and ACP docking site of *Streptomyces coelicolor* Malonyl-CoA:ACP transacylase. *Structure* **11**, 147–154 (2003).
16. Wong, F. T., Jin, X., Mathews, I. I., Cane, D. E. & Khosla, C. Structure and mechanism of the *trans*-acting acyltransferase from the disorazole synthase. *Biochemistry* **50**, 6539–6548 (2011).
17. Liew, C. W. et al. Crystal structure of the acyltransferase domain of the iterative polyketide synthase in enediyne biosynthesis. *J. Biol. Chem.* **287**, 23203–23215 (2012).
18. Zheng, J., Taylor, C. A., Piasecki, S. K. & Keatinge-Clay, A. T. Structural and functional analysis of A-type ketoreductases from the amphotericin modular polyketide synthase. *Structure* **18**, 913–922 (2010).
19. Zheng, J., Piasecki, S. K. & Keatinge-Clay, A. T. Structural studies of an A2-type modular polyketide synthase ketoreductase reveal features controlling α-substituent stereochemistry. *ACS Chem. Biol.* **8**, 1964–1971 (2013).
20. Keatinge-Clay, A. T. A tylosin ketoreductase reveals how chirality is determined in polyketides. *Chem. Biol.* **14**, 898–908 (2007).
21. Keatinge-Clay, A. T. & Stroud, R. M. The structure of a ketoreductase determines the organization of the β-carbon processing enzymes of modular polyketide synthases. *Structure* **14**, 737–748 (2006).
22. Zheng, J. & Keatinge-Clay, A. T. Structural and functional analysis of C2-type ketoreductases from modular polyketide synthases. *J. Mol. Biol.* **410**, 105–117 (2011).
23. Gay, D. C. et al. A close look at a ketosynthase from a *trans*-acyltransferase modular polyketide synthase. *Structure* **22**, 444–451 (2014).
24. Du, D. et al. Structural basis for selectivity in a highly reducing type II polyketide synthase. *Nat. Chem. Biol.* **16**, 776–782 (2020).
25. Bräuer, A. et al. Structural snapshots of the minimal PKS system responsible for octaketide biosynthesis. *Nat. Chem.* **12**, 755–763 (2020).
26. Kieser, T., Bibb, M. J., Buttner, M. J., Chater, K. F., Hopwood, D. A., *Practical Streptomyces Genetics*. (Norwich: The John Innes Foundation., 2000).
27. Wu, Q. et al. Characterization of the biosynthesis gene cluster for the pyrrole polyether antibiotic calcimycin (A23187) in *Streptomyces chartreusis* NRRL 3882. *Antimicrob. Agents Chemother.* **55**, 974–982 (2011).

28. Huang, T. et al. Identification and characterization of the pyridomycin biosynthetic gene cluster of *Streptomyces pyridomyceticus* NRRL B-2517. *J. Biol. Chem.* **286**, 20648–20657 (2011).
